# Supplementary material for: Automatic approach-avoidance tendency toward physical activity, sedentary, and neutral stimuli as a function of age, explicit affective attitude, and intention to be active
Source: Peer Community J. Author manuscript; Available in PMC 2024 Dec 10. (PMC7617180; doi:10.24072/pcjournal.246)
Supplement: Data, code, and supplemental material [file EMS201322-supplement-Data__code__and_supplemental_material.zip › Boisgontier-Lab-Aging_Approach-Avoid_Physical-Activity-0a8d854/Materials/Consent_Form_FR.html]

**Formulaire de Consentement**

Veuillez s’il vous plait lire attentivement ce document de consentement avant de décider de participer à cette étude. 

 

**Titre de l’étude :** Processus automatiques associés aux comportements d'activité physique et de sédentarité tout au long de la vie.

 

**Objectif de l'étude :** L'objectif de l'étude est d'améliorer notre compréhension des mécanismes qui sous-tendent l'engagement dans l'activité physique tout au long de la vie.

 

**Participation :** Ma participation à cette étude en ligne de 30 minutes consistera à répondre à des questionnaires et à effectuer une tâche de temps de réaction sur mon ordinateur à l'aide du logiciel Inquisit. Les questionnaires comprennent des questions relatives à des informations démographiques, aux ressources de maîtrise de soi, à la perception du poids, à la satisfaction corporelle et à l'apathie. Dans la tâche de temps de réaction, on me demandera d’approcher ou d’écarter un mannequin d’images présentées sur l’écran de mon ordinateur en appuyant sur les touches du clavier.

 

**Risques :** Ma participation à cette étude ne comporte aucun risque et aucune gêne autres que ceux de la vie quotidienne. Cependant, si je ressens un quelconque inconfort pendant l'étude, je peux arrêter à tout moment en cliquant sur la combinaison de touches "ctrl + Q" pour quitter l'expérience.

 

**Avantages :** Ma participation à cette étude contribuera à mieux comprendre les facteurs qui facilitent ou inhibent l'engagement dans une activité physique, aidant ainsi les individus à adopter un mode de vie plus actif et plus sain. Ma participation à ce projet aidera également les étudiants en master de physiothérapie de l’Université d’Ottawa à développer leurs connaissances et leurs compétences en matière de recherche scientifique. 

 

**Confidentialité et vie privée :** Mon anonymat sera garanti par un code d'identification confidentiel unique. Mes réponses aux questionnaires resteront strictement confidentielles. Mon nom et mes coordonnées ne seront pas recueillis et mes réponses sont donc anonymes.

 

**Conservation des données :** Les données recueillies par le biais du site Web d'Inquisit seront conservées dans les installations du centre de données de classe mondiale d'Inquisit dans l'Oregon, aux États-Unis, et ne seront pas déplacées vers d'autres endroits. Pendant l’analyse de données, ces dernières seront stockées sur le compte Microsoft OneDrive de l'Université d'Ottawa du chercheur principal qui est protégé par une authentification à deux facteurs.

 

**Participation volontaire :** Je n'ai aucune obligation de participer et si je choisis de participer, je peux me retirer de l'étude à tout moment et refuser de répondre à toute question, sans subir de conséquences négatives. Si je choisis de me retirer, je peux contacter le chercheur principal et lui demander que les données relatives à mon code d'identification confidentiel unique soient supprimées. Toutes les données recueillies jusqu'au moment du retrait seront alors supprimées de l'ensemble de données et ne seront pas utilisées dans l'étude. 

 

**Accord :** J'ai lu la description de l’étude ci-dessus et j'accepte volontairement de participer.

 

Si j'ai des questions concernant l'étude, je peux contacter le chercheur principal du projet aux coordonnées ci-dessous. Si j'ai des questions concernant l’éthique de cette étude, je peux contacter le Bureau de l'éthique et de l'intégrité de la recherche par courriel (ethics@uottawa.ca) ou par téléphone (613-562-5387). Pour obtenir une copie de ce consentement et obtenir des renseignements supplémentaires sur vos droits en tant que participant à l'étude, je peux envoyer un courriel au Dr Matthieu Boisgontier (matthieu.boisgontier@uottawa.ca) à l'École des sciences de la réadaptation, Faculté des sciences de la santé, Université d'Ottawa. Les participants sont encouragés à conserver une copie du formulaire de consentement.

 

**Acceptation :** En choisissant la phrase ci-dessous, je consens ou ne consens pas à participer à cette recherche.

 

Oui, je veux participer.

Non, je ne veux pas participer.
