# Supplementary material for: Automatic approach-avoidance tendency toward physical activity, sedentary, and neutral stimuli as a function of age, explicit affective attitude, and intention to be active
Source: Peer Community J. Author manuscript; Available in PMC 2024 Dec 10. (PMC7617180; doi:10.24072/pcjournal.246)
Supplement: Data, code, and supplemental material [file EMS201322-supplement-Data__code__and_supplemental_material.zip › Boisgontier-Lab-Aging_Approach-Avoid_Physical-Activity-0a8d854/Materials/Inquisit code_FR.docx]

Code for the French version of the approach avoidance task on Inquisit 6 (Millisecond Software, Version 6.5.2, <https://www.millisecond.com>)

<usermanual>

LIFESPAN STUDY (ENGLISH VERSION)

</usermanual>

<parameters>

/ responsekey_up = 22

/ responsekey_down = 49

/ upkeylabel = "U"

/ downkeylabel = "N"

</parameters>

<instruct>

/ fontstyle = ("Arial", 3%, false, false, false, false, 5, 1)

/ txcolor = black

/ lastlabel = ("Press the space bar to continue")

/ nextlabel = ("Press the space bar to continue")

</instruct>

<defaults >

/canvasaspectratio = (4,3)

/ minimumversion = "6.5.0.0"

/ fontstyle = ("Arial", 3%, false, false, false, false, 5, 1)

/txbgcolor = white

/ txcolor = (0, 0, 0)

/ inputdevice = keyboard

</defaults>

********************

Output of raw data

********************

<data>

/ separatefiles = true

/ columns = (textbox.participant.response, build, computer.platform, date, time, subject, group, blockcode, blocknum, trialcode, latency, correct, trialnum, response, script.elapsedtime,

parameters.responsekey_up, parameters.responsekey_down, values.ap_pictureindex, values.seden_pictureindex, values.circle_pictureindex, values.square_pictureindex,textbox.age.response,textbox.height.response,textbox.weight.response,radiobuttons.sex.response,radiobuttons.gender.response,textbox.country.response,checkboxes.chronic.response,

textbox.vigorous_d.response,textbox.vigorous_m.response,textbox.moderate_d.response,textbox.moderate_m.response,textbox.sedentary_d.response,textbox.sedentary_m.response,radiobuttons.intention.response,radiobuttons.attitude1.response,radiobuttons.attitude2.response,

expressions.propcorr_ApApproach, expressions.meanRT_ApApproach,

expressions.propcorr_ApAvoid, expressions.meanRT_ApAvoid,

expressions.propcorr_SedenApproach, expressions.meanRT_SedenApproach,

expressions.propcorr_SedenAvoid, expressions.meanRT_SedenAvoid,

expressions.propcorr_circleApproach, expressions.meanRT_circleApproach,

expressions.propcorr_circleAvoid, expressions.meanRT_circleAvoid,

expressions.propcorr_squareApproach, expressions.meanRT_squareApproach,

expressions.propcorr_squareAvoid, expressions.meanRT_squareAvoid)

</data>

************************

Approach-Avoidance Task

************************

<values>

/ completed = 0

/ ap_pictureindex = 0

/ seden_pictureindex = 0

/ circle_pictureindex = 0

/ square_pictureindex = 0

/ nextpicture = 0

/ selectap = 0

/ selectseden = 0

/ stimulus = ""

/ test = 0

</values>

*****

<expressions >

/ propcorr_total = list.accuracy_alltrials.mean

/ meanRT_total = list.latencies_allcorrecttrials.mean

/ propcorr_ApApproach = list.accuracy_ApproachAp.mean

/ meanRT_ApApproach = list.latencies_corrApproachAp.mean

/ propcorr_ApAvoid = list.accuracy_AvoidAp.mean

/ meanRT_ApAvoid = list.latencies_corrAvoidAp.mean

/ propcorr_SedenApproach = list.accuracy_ApproachSeden.mean

/ meanRT_SedenApproach = list.latencies_corrApproachSeden.mean

/ propcorr_SedenAvoid = list.accuracy_AvoidSeden.mean

/ meanRT_SedenAvoid = list.latencies_corrAvoidSeden.mean

/ propcorr_circleApproach = list.accuracy_ApproachCircle.mean

/ meanRT_circleApproach = list.latencies_corrApproachCircle.mean

/ propcorr_circleAvoid = list.accuracy_AvoidCircle.mean

/ meanRT_circleAvoid = list.latencies_corrAvoidCircle.mean

/ propcorr_squareApproach = list.accuracy_ApproachSquare.mean

/ meanRT_squareApproach = list.latencies_corrApproachSquare.mean

/ propcorr_squareAvoid = list.accuracy_AvoidSquare.mean

/ meanRT_squareAvoid = list.latencies_corrAvoidSquare.mean

</expressions>

<expressions>

/fixduration = rand(500,750)

</expressions>

<picture ap>

/ items = appics

/ position = (50%,50%)

/ erase = false

</picture>

<picture seden>

/ items = sedenpics

/ position = (50%,50%)

/ erase = false

</picture>

<picture circle>

/ items = circlepics

/ position = (50%,50%)

/ size = (20%, 20%)

/ erase = false

</picture>

<picture square>

/ items = squarepics

/ position = (50%,50%)

/ size = (20%, 20%)

/ erase = false

</picture>

*********

<page intro>

^Merci de votre participation à cette étude en ligne qui sera divisée en 3 parties :

^^1- Consentement éclairé et questions démographiques.

^^2- Tâche dite "du mannequin" dans 4 conditions différentes.

^^3- Questionnaires.

^^Vous aurez à répondre à toutes les questions pour pouvoir passer à la page suivante en cliquant sur le bouton "Suivant" situé en bas de la page.

^^Veuillez appuyer sur la barre espace de votre clavier lorsque vous êtes prêt à commencer.

</page>

<page end>

^^Merci pour votre participation :)

</page>

*** Manikin instructions

<page approach_circle>

^^Localisez les touches "<%parameters.upkeylabel%>" et "<%parameters.downkeylabel%>" de votre clavier. Ces 2 touches vont être utiles ici.

^^Dans la tâche qui suit, un mannequin qui VOUS représente apparaîtra soit en bas de l'écran, soit en haut.

^^Ensuite, une image apparaîtra au centre l'écran.

^^Votre tâche est de déplacer le mannequin aussi vite que possiblevers les cercles et d'éviter les carrés.

^^ - Si l'image représente des CERCLES et des OVALES, vous devez APPROCHER le mannequin du cercle (APPROCHE CERCLES).

^^ - Si l'image représente des CARRES et des RECTANGLES, vous devez ELOIGNER le mannequin du carré (EVITEMENT CARRES).

^^Appuyez sur la touche "<%parameters.upkeylabel%>" qui permet de MONTER le mannequin vers le haut et la touche "<%parameters.downkeylabel%>" qui permet de le DESCENDRE vers le bas, en utilisant vos 2 indexs.

^^Appuyez sur la BARRE ESPACE pour commencer la session d'essai.

</page>

<page approach_ap>

^^Vous pouvez commencer par bien localiser les touches "<%parameters.upkeylabel%>" et "<%parameters.downkeylabel%>" de votre clavier. Ces 2 touches vont vous être utiles.

^^Dans la tâche qui suit, un mannequin qui VOUS représente apparaîtra soit en bas de l'écran, soit en haut.

^^Ensuite, une image apparaîtra au centre l'écran.

^^Votre tâche est de déplacer le mannequin aussi vite que possible vers les images d'activité physique et d'éviter les images de sédentarité.

^^ - Si l'image représente une activité PHYSIQUE, vous devez APPROCHER le mannequin de l'image (APPROCHE ACTIVITE PHYSIQUE).

^^ - Si l'image représente une activité SEDENTAIRE, vous devez ELOIGNER le mannequin de l'image (EVITEMENT SEDENTARITE).

^^Appuyez sur la touche "<%parameters.upkeylabel%>" qui permet de MONTER le mannequin vers le haut et la touche "<%parameters.downkeylabel%>" qui permet de le DESCENDRE vers le bas, en utilisant vos 2 indexs.

^^Appuyez sur la BARRE ESPACE pour commencer la session d'essai.

</page>

<page avoid_circle>

^^Vous pouvez commencer par bien localiser les touches "<%parameters.upkeylabel%>" et "<%parameters.downkeylabel%>" de votre clavier. Ces 2 touches vont vous être utiles.

^^Dans la tâche qui suit, un mannequin qui VOUS représente apparaîtra soit en bas de l'écran, soit en haut.

^^Ensuite, une image apparaîtra au centre l'écran.

^^Votre tâche est de déplacer le mannequin aussi vite que possible vers les carrés et d'éviter les cercles.

^^ - Si l'image représente des CERCLES et des OVALES, vous devez ELOIGNER le mannequin de l'image (EVITEMENT CERCLES).

^^ - Si l'image représente des CARRES et des RECTANGLES, vous devez APPROCHER le mannequin de l'image (APPROCHE CARRES).

^^Appuyez sur la touche "<%parameters.upkeylabel%>" qui permet de MONTER le mannequin vers le haut et la touche "<%parameters.downkeylabel%>" qui permet de le DESCENDRE vers le bas, en utilisant vos 2 indexs.

^^Appuyez sur la BARRE ESPACE pour commencer la session d'essai.

</page>

<page avoid_ap>

^^Vous pouvez commencer par bien localiser les touches "<%parameters.upkeylabel%>" et "<%parameters.downkeylabel%>" de votre clavier. Ces 2 touches vont vous être utiles.

^^Dans la tâche qui suit, un mannequin qui VOUS représente apparaîtra soit en bas de l'écran, soit en haut.

^^Ensuite, une image apparaîtra au centre l'écran.

^^Votre tâche est de déplacer le mannequin aussi vite que possible vers les images de sédentarité et d'éviter les images d'activité physique.

^^ - Si l'image représente une activité PHYSIQUE, vous devez ELOIGNER le mannequin de l'image (EVITEMENT ACTIVITE PHYSIQUE).

^^ - Si l'image représente une activité SEDENTAIRE, vous devez APPROCHER le mannequin de l'image (APPROCHE SEDENTARITE).

^^Appuyez sur la touche "<%parameters.upkeylabel%>" qui permet de MONTER le mannequin vers le haut et la touche "<%parameters.downkeylabel%>" qui permet de le DESCENDRE vers le bas, en utilisant vos 2 indexs.

^^Appuyez sur la BARRE ESPACE pour commencer la session d'essai.

</page>

........

<text fixation>

/ items = ("+")

/ fontstyle = ("Arial", 5.00%, true)

</text>

<text reminder>

/ items = ("Congratulations on completing this part of the study.

Please read the following message carefully as the instructions change.

~nPlease press the space bar to continue")

/ fontstyle = ("Arial", 3.00%, true)

</text>

<picture manikintop>

/ items = ("manikin_2.jpg")

/ position = (50%, 20%)

</picture>

<picture manikin_top_moveup>

/ items = ("manikin_2.jpg")

/ position = (50%, 10%)

</picture>

<picture manikin_top_movedown>

/ items = ("manikin_2.jpg")

/ position = (50%, 30%)

</picture>

<picture manikinbottom>

/ items = ("manikin_2.jpg")

/ position = (50%, 80%)

</picture>

<picture manikin_bottom_moveup>

/ items = ("manikin_2.jpg")

/ position = (50%, 70%)

</picture>

<picture manikin_bottom_movedown>

/ items = ("manikin_2.jpg")

/ position = (50%, 90%)

</picture>

<text error>

/ fontstyle = ("arial", 22pt, true)

/ items = ("ERROR")

/ position = (50%,50%)

/ txcolor = (red)

/ erase = false

</text>

<text trop_lent>

/ fontstyle = ("arial", 22pt, true)

/ items = ("TOO SLOW")

/ position = (50%,50%)

/ txcolor = (red)

/ erase = false

</text>

<picture instructionimages>

/ items = ("instructionimages.png")

/ position = (50%,50%)

/ size = (80%, 80%)

</picture>

Note: the following 4 lists help determine which pictures are presented during the experimental trials

<list ap_pictureindex>

/ items = (1, 2, 3, 4, 5, 6)

/ selectionrate = trial

/ selectionmode = random

</list>

<list seden_pictureindex>

/ items = (7, 8, 9, 10, 11, 12)

/ selectionrate = trial

/ selectionmode = random

</list>

<list circle_pictureindex>

/ items = (13,14,15,16,17,18)

/ selectionrate = trial

/ selectionmode = random

</list>

<list square_pictureindex>

/ items = (19,20,21,22,23,24)

/ selectionrate = trial

/ selectionmode = random

</list>

*************************************************

Data Lists: used for descriptive statistics

store latencies/accuracy data

fill up during runtime

*************************************************

<list accuracy_alltrials>

</list>

<list latencies_allcorrecttrials>

</list>

<list accuracy_ApproachAp>

</list>

<list latencies_corrApproachAp>

</list>

<list accuracy_AvoidAp>

</list>

<list latencies_corrAvoidAp>

</list>

<list accuracy_ApproachSeden>

</list>

<list latencies_corrApproachSeden>

</list>

<list accuracy_AvoidSeden>

</list>

<list latencies_corrAvoidSeden>

</list>

<list accuracy_ApproachCircle>

</list>

<list latencies_corrApproachCircle>

</list>

<list accuracy_AvoidCircle>

</list>

<list latencies_corrAvoidCircle>

</list>

<list accuracy_ApproachSquare>

</list>

<list latencies_corrApproachSquare>

</list>

<list accuracy_AvoidSquare>

</list>

<list latencies_corrAvoidSquare>

</list>

............

<item appics>

/ 1 = "AP-COUR.jpg"

/ 2 = "AP-ESCAL.jpg"

/ 3 = "AP-FOOT.jpg"

/ 4 = "AP-NAT.jpg"

/ 5 = "AP-RANDO.jpg"

/ 6 = "AP-VEL.jpg"

</item>

<item sedenpics>

/ 7 = "SED-CANAP.jpg"

/ 8 = "SED-HAMAC.jpg"

/ 9 = "SED-JVID.jpg"

/ 10 = "SED-LECT.jpg"

/ 11 = "SED-ORDI.jpg"

/ 12 = "SED-TV.jpg"

</item>

<item circlepics>

/ 13 = "AP-NATr.jpg"

/ 14 = "AP-RANDOr.jpg"

/ 15 = "AP-VELr.jpg"

/ 16 = "SED-CANAPr.jpg"

/ 17 = "SED-HAMACr.jpg"

/ 18 = "SED-LECTr.jpg"

</item>

<item squarepics>

/ 19 = "AP-NATc.jpg"

/ 20 = "AP-RANDOc.jpg"

/ 21 = "AP-VELc.jpg"

/ 22 = "SED-CANAPc.jpg"

/ 23 = "SED-HAMACc.jpg"

/ 24 = "SED-LECTc.jpg"

</item>

........

........

<trial error>

/ trialcode = "error"

/ stimulustimes = [0= error]

/ trialduration = (800)

/ responsemode = noresponse

</trial>

<trial trop_lent>

/ trialcode = "too_slow"

/ stimulustimes = [0= trop_lent]

/ trialduration = (800)

/ responsemode = noresponse

</trial>

<trial instructionimages>

/trialcode = "instructionimages"

/ stimulustimes = [0=instructionimages]

/ trialduration = 7000

</trial>

*******physical activity picture man top approach: 1

<trial ApApproach_ManikinTop>

/ ontrialbegin = [values.ap_pictureindex = list.ap_pictureindex.nextvalue]

/ stimulustimes = [0 = clearscreen, manikintop; 1000=ap]

/ validresponse = (parameters.responsekey_up, parameters.responsekey_down)

/ correctresponse = (parameters.responsekey_down)

/ ontrialend = [

if (values.test == 1) {

list.accuracy_alltrials.insertitem(trial.ApApproach_ManikinTop.correct, 1);

list.accuracy_ApproachAp.insertitem(trial.ApApproach_ManikinTop.correct, 1);

if (trial.ApApproach_ManikinTop.correct) {

list.latencies_allcorrecttrials.insertitem(trial.ApApproach_ManikinTop.latency, 1);

list.latencies_corrApproachAp.insertitem(trial.ApApproach_ManikinTop.latency, 1);

}

}

]

/ correctmessage = (manikin_top_movedown, 1000)

/ branch = [if (trial.ApApproach_ManikinTop.response == 0) trial.trop_lent]

/ branch = [if (trial.ApApproach_ManikinTop.error) trial.error]

/ timeout = 8000

</trial>

*******physical activity picture man bottom approach: 2

<trial ApApproach_ManikinBottom>

/ ontrialbegin = [values.ap_pictureindex = list.ap_pictureindex.nextvalue]

/ stimulustimes = [0 = clearscreen, manikinbottom; 1000=ap]

/ validresponse = (parameters.responsekey_up, parameters.responsekey_down)

/ correctresponse = (parameters.responsekey_up)

/ ontrialend = [

if (values.test == 1) {

list.accuracy_alltrials.insertitem(trial.ApApproach_ManikinBottom.correct, 1);

list.accuracy_ApproachAp.insertitem(trial.ApApproach_ManikinBottom.correct, 1);

if (trial.ApApproach_ManikinBottom.correct) {

list.latencies_allcorrecttrials.insertitem(trial.ApApproach_ManikinBottom.latency, 1);

list.latencies_corrApproachAp.insertitem(trial.ApApproach_ManikinBottom.latency, 1);

}

}

]

/ branch = [if (trial.ApApproach_ManikinBottom.response == 0) trial.trop_lent]

/ branch = [if (trial.ApApproach_ManikinBottom.error) trial.error]

/ correctmessage = (manikin_bottom_moveup, 1000)

/ timeout = 8000

</trial>

*******physical activity picture man top avoid: 3

<trial ApAvoid_ManikinTop>

/ ontrialbegin = [values.ap_pictureindex = list.ap_pictureindex.nextvalue]

/ stimulustimes = [0 = clearscreen, manikintop; 1000=ap]

/ validresponse = (parameters.responsekey_up, parameters.responsekey_down)

/ correctresponse = (parameters.responsekey_up)

/ ontrialend = [

if (values.test == 1) {

list.accuracy_alltrials.insertitem(trial.ApAvoid_ManikinTop.correct, 1);

list.accuracy_AvoidAp.insertitem(trial.ApAvoid_ManikinTop.correct, 1);

if (trial.ApAvoid_ManikinTop.correct) {

list.latencies_allcorrecttrials.insertitem(trial.ApAvoid_ManikinTop.latency, 1);

list.latencies_corrAvoidAp.insertitem(trial.ApAvoid_ManikinTop.latency, 1);

}

}

]

/ branch = [if (trial.ApAvoid_ManikinTop.response == 0) trial.trop_lent]

/ branch = [if (trial.ApAvoid_ManikinTop.error) trial.error]

/ correctmessage = (manikin_top_moveup, 1000)

/ timeout = 8000

</trial>

*******physical activity picture man bottom avoid: 4

<trial ApAvoid_ManikinBottom>

/ ontrialbegin = [values.ap_pictureindex = list.ap_pictureindex.nextvalue]

/ stimulustimes = [0 = clearscreen, manikinbottom; 1000=ap]

/ validresponse = (parameters.responsekey_up, parameters.responsekey_down)

/ correctresponse = (parameters.responsekey_down)

/ ontrialend = [

if (values.test == 1) {

list.accuracy_alltrials.insertitem(trial.ApAvoid_ManikinBottom.correct, 1);

list.accuracy_AvoidAp.insertitem(trial.ApAvoid_ManikinBottom.correct, 1);

if (trial.ApAvoid_ManikinBottom.correct) {

list.latencies_allcorrecttrials.insertitem(trial.ApAvoid_ManikinBottom.latency, 1);

list.latencies_corrAvoidAp.insertitem(trial.ApAvoid_ManikinBottom.latency, 1);

}

}

]

/ branch = [if (trial.ApAvoid_ManikinBottom.response == 0) trial.trop_lent]

/ branch = [if (trial.ApAvoid_ManikinBottom.error) trial.error]

/ correctmessage = (manikin_bottom_movedown, 1000)

/ timeout = 8000

</trial>

*******sedentary picture man top approach: 5

<trial SedenApproach_ManikinTop>

/ ontrialbegin = [values.seden_pictureindex = list.seden_pictureindex.nextvalue]

/ stimulustimes = [0 = clearscreen, manikintop; 1000=seden]

/ validresponse = (parameters.responsekey_up, parameters.responsekey_down)

/ correctresponse = (parameters.responsekey_down)

/ ontrialend = [

if (values.test == 1) {

list.accuracy_alltrials.insertitem(trial.SedenApproach_ManikinTop.correct, 1);

list.accuracy_ApproachSeden.insertitem(trial.SedenApproach_ManikinTop.correct, 1);

if (trial.SedenApproach_ManikinTop.correct) {

list.latencies_allcorrecttrials.insertitem(trial.SedenApproach_ManikinTop.latency, 1);

list.latencies_corrApproachSeden.insertitem(trial.SedenApproach_ManikinTop.latency, 1);

}

}

]

/ branch = [if (trial.SedenApproach_ManikinTop.response == 0) trial.trop_lent]

/ branch = [if (trial.SedenApproach_ManikinTop.error) trial.error]

/ correctmessage = (manikin_top_movedown, 1000)

/ timeout = 8000

</trial>

*******sedentary picture man bottom approach: 6

<trial SedenApproach_ManikinBottom>

/ ontrialbegin = [values.seden_pictureindex = list.seden_pictureindex.nextvalue]

/ stimulustimes = [0=clearscreen, manikinbottom; 1000=seden]

/ validresponse = (parameters.responsekey_up, parameters.responsekey_down)

/ correctresponse = (parameters.responsekey_up)

/ ontrialend = [

if (values.test == 1) {

list.accuracy_alltrials.insertitem(trial.SedenApproach_ManikinBottom.correct, 1);

list.accuracy_ApproachSeden.insertitem(trial.SedenApproach_ManikinBottom.correct, 1);

if (trial.SedenApproach_ManikinBottom.correct) {

list.latencies_allcorrecttrials.insertitem(trial.SedenApproach_ManikinBottom.latency, 1);

list.latencies_corrApproachSeden.insertitem(trial.SedenApproach_ManikinBottom.latency, 1);

}

}

]

/ branch = [if (trial.SedenApproach_ManikinBottom.response == 0) trial.trop_lent]

/ branch = [if (trial.SedenApproach_ManikinBottom.error) trial.error]

/ correctmessage = (manikin_bottom_moveup, 1000)

/ timeout = 8000

</trial>

*******sedentaryt picture man top avoid: 7

<trial SedenAvoid_ManikinTop>

/ ontrialbegin = [values.seden_pictureindex = list.seden_pictureindex.nextvalue]

/ stimulustimes = [0 = clearscreen, manikintop; 1000=seden]

/ validresponse = (parameters.responsekey_up, parameters.responsekey_down)

/ correctresponse = (parameters.responsekey_up)

/ ontrialend = [

if (values.test == 1) {

list.accuracy_alltrials.insertitem(trial.SedenAvoid_ManikinTop.correct, 1);

list.accuracy_AvoidSeden.insertitem(trial.SedenAvoid_ManikinTop.correct, 1);

if (trial.SedenAvoid_ManikinTop.correct) {

list.latencies_allcorrecttrials.insertitem(trial.SedenAvoid_ManikinTop.latency, 1);

list.latencies_corrAvoidSeden.insertitem(trial.SedenAvoid_ManikinTop.latency, 1);

}

}

]

/ branch = [if (trial.SedenAvoid_ManikinTop.response == 0) trial.trop_lent]

/ branch = [if (trial.SedenAvoid_ManikinTop.error) trial.error]

/ correctmessage = (manikin_top_moveup, 1000)

/ timeout = 8000

</trial>

*******sedentary picture man bottom avoid: 8

<trial SedenAvoid_ManikinBottom>

/ ontrialbegin = [values.seden_pictureindex = list.seden_pictureindex.nextvalue]

/ stimulustimes = [0=clearscreen, manikinbottom; 1000=seden]

/ validresponse = (parameters.responsekey_up, parameters.responsekey_down)

/ correctresponse = (parameters.responsekey_down)

/ ontrialend = [

if (values.test == 1) {

list.accuracy_alltrials.insertitem(trial.SedenAvoid_ManikinBottom.correct, 1);

list.accuracy_AvoidSeden.insertitem(trial.SedenAvoid_ManikinBottom.correct, 1);

if (trial.SedenAvoid_ManikinBottom.correct) {

list.latencies_allcorrecttrials.insertitem(trial.SedenAvoid_ManikinBottom.latency, 1);

list.latencies_corrAvoidSeden.insertitem(trial.SedenAvoid_ManikinBottom.latency, 1);

}

}

]

/ branch = [if (trial.SedenAvoid_ManikinBottom.response == 0) trial.trop_lent]

/ branch = [if (trial.SedenAvoid_ManikinBottom.error) trial.error]

/ correctmessage = (manikin_bottom_movedown, 1000)

/ timeout = 8000

</trial>

*******circle picture man top approach: 1

<trial circleApproach_ManikinTop>

/ ontrialbegin = [values.circle_pictureindex = list.circle_pictureindex.nextvalue]

/ stimulustimes = [0 = clearscreen, manikintop; 1000=circle]

/ validresponse = (parameters.responsekey_up, parameters.responsekey_down)

/ correctresponse = (parameters.responsekey_down)

/ ontrialend = [

if (values.test == 1) {

list.accuracy_alltrials.insertitem(trial.circleApproach_ManikinTop.correct, 1);

list.accuracy_ApproachCircle.insertitem(trial.circleApproach_ManikinTop.correct, 1);

if (trial.circleApproach_ManikinTop.correct) {

list.latencies_allcorrecttrials.insertitem(trial.circleApproach_ManikinTop.latency, 1);

list.latencies_corrApproachCircle.insertitem(trial.circleApproach_ManikinTop.latency, 1);

}

}

]

/ correctmessage = (manikin_top_movedown, 1000)

/ branch = [if (trial.circleApproach_ManikinTop.response == 0) trial.trop_lent]

/ branch = [if (trial.circleApproach_ManikinTop.error) trial.error]

/ timeout = 8000

</trial>

*******circle picture man bottom approach: 2

<trial circleApproach_ManikinBottom>

/ ontrialbegin = [values.circle_pictureindex = list.circle_pictureindex.nextvalue]

/ stimulustimes = [0 = clearscreen, manikinbottom; 1000=circle]

/ validresponse = (parameters.responsekey_up, parameters.responsekey_down)

/ correctresponse = (parameters.responsekey_up)

/ ontrialend = [

if (values.test == 1) {

list.accuracy_alltrials.insertitem(trial.circleApproach_ManikinBottom.correct, 1);

list.accuracy_ApproachCircle.insertitem(trial.circleApproach_ManikinBottom.correct, 1);

if (trial.circleApproach_ManikinBottom.correct) {

list.latencies_allcorrecttrials.insertitem(trial.circleApproach_ManikinBottom.latency, 1);

list.latencies_corrApproachCircle.insertitem(trial.circleApproach_ManikinBottom.latency, 1);

}

}

]

/ branch = [if (trial.circleApproach_ManikinBottom.response == 0) trial.trop_lent]

/ branch = [if (trial.circleApproach_ManikinBottom.error) trial.error]

/ correctmessage = (manikin_bottom_moveup, 1000)

/ timeout = 8000

</trial>

*******circle picture man top avoid: 3

<trial circleAvoid_ManikinTop>

/ ontrialbegin = [values.circle_pictureindex = list.circle_pictureindex.nextvalue]

/ stimulustimes = [0 = clearscreen, manikintop; 1000=circle]

/ validresponse = (parameters.responsekey_up, parameters.responsekey_down)

/ correctresponse = (parameters.responsekey_up)

/ ontrialend = [

if (values.test == 1) {

list.accuracy_alltrials.insertitem(trial.circleAvoid_ManikinTop.correct, 1);

list.accuracy_AvoidCircle.insertitem(trial.circleAvoid_ManikinTop.correct, 1);

if (trial.circleAvoid_ManikinTop.correct) {

list.latencies_allcorrecttrials.insertitem(trial.circleAvoid_ManikinTop.latency, 1);

list.latencies_corrAvoidCircle.insertitem(trial.circleAvoid_ManikinTop.latency, 1);

}

}

]

/ branch = [if (trial.circleAvoid_ManikinTop.response == 0) trial.trop_lent]

/ branch = [if (trial.circleAvoid_ManikinTop.error) trial.error]

/ correctmessage = (manikin_top_moveup, 1000)

/ timeout = 8000

</trial>

*******circle picture man bottom avoid: 4

<trial circleAvoid_ManikinBottom>

/ ontrialbegin = [values.circle_pictureindex = list.circle_pictureindex.nextvalue]

/ stimulustimes = [0 = clearscreen, manikinbottom; 1000=circle]

/ validresponse = (parameters.responsekey_up, parameters.responsekey_down)

/ correctresponse = (parameters.responsekey_down)

/ ontrialend = [

if (values.test == 1) {

list.accuracy_alltrials.insertitem(trial.circleAvoid_ManikinBottom.correct, 1);

list.accuracy_AvoidCircle.insertitem(trial.circleAvoid_ManikinBottom.correct, 1);

if (trial.circleAvoid_ManikinBottom.correct) {

list.latencies_allcorrecttrials.insertitem(trial.circleAvoid_ManikinBottom.latency, 1);

list.latencies_corrAvoidCircle.insertitem(trial.circleAvoid_ManikinBottom.latency, 1);

}

}

]

/ branch = [if (trial.circleAvoid_ManikinBottom.response == 0) trial.trop_lent]

/ branch = [if (trial.circleAvoid_ManikinBottom.error) trial.error]

/ correctmessage = (manikin_bottom_movedown, 1000)

/ timeout = 8000

</trial>

*******square picture man top approach: 5

<trial squareApproach_ManikinTop>

/ ontrialbegin = [values.square_pictureindex = list.square_pictureindex.nextvalue]

/ stimulustimes = [0 = clearscreen, manikintop; 1000=square]

/ validresponse = (parameters.responsekey_up, parameters.responsekey_down)

/ correctresponse = (parameters.responsekey_down)

/ ontrialend = [

if (values.test == 1) {

list.accuracy_alltrials.insertitem(trial.squareApproach_ManikinTop.correct, 1);

list.accuracy_ApproachSquare.insertitem(trial.squareApproach_ManikinTop.correct, 1);

if (trial.squareApproach_ManikinTop.correct) {

list.latencies_allcorrecttrials.insertitem(trial.squareApproach_ManikinTop.latency, 1);

list.latencies_corrApproachSquare.insertitem(trial.squareApproach_ManikinTop.latency, 1);

}

}

]

/ branch = [if (trial.squareApproach_ManikinTop.response == 0) trial.trop_lent]

/ branch = [if (trial.squareApproach_ManikinTop.error) trial.error]

/ correctmessage = (manikin_top_movedown, 1000)

/ timeout = 8000

</trial>

*******square picture man bottom approach: 6

<trial squareApproach_ManikinBottom>

/ ontrialbegin = [values.square_pictureindex = list.square_pictureindex.nextvalue]

/ stimulustimes = [0=clearscreen, manikinbottom; 1000=square]

/ validresponse = (parameters.responsekey_up, parameters.responsekey_down)

/ correctresponse = (parameters.responsekey_up)

/ ontrialend = [

if (values.test == 1) {

list.accuracy_alltrials.insertitem(trial.squareApproach_ManikinBottom.correct, 1);

list.accuracy_ApproachSquare.insertitem(trial.squareApproach_ManikinBottom.correct, 1);

if (trial.squareApproach_ManikinBottom.correct) {

list.latencies_allcorrecttrials.insertitem(trial.squareApproach_ManikinBottom.latency, 1);

list.latencies_corrApproachSquare.insertitem(trial.squareApproach_ManikinBottom.latency, 1);

}

}

]

/ branch = [if (trial.squareApproach_ManikinBottom.response == 0) trial.trop_lent]

/ branch = [if (trial.squareApproach_ManikinBottom.error) trial.error]

/ correctmessage = (manikin_bottom_moveup, 1000)

/ timeout = 8000

</trial>

*******square picture man top avoid: 7

<trial squareAvoid_ManikinTop>

/ ontrialbegin = [values.square_pictureindex = list.square_pictureindex.nextvalue]

/ stimulustimes = [0 = clearscreen, manikintop; 1000=square]

/ validresponse = (parameters.responsekey_up, parameters.responsekey_down)

/ correctresponse = (parameters.responsekey_up)

/ ontrialend = [

if (values.test == 1) {

list.accuracy_alltrials.insertitem(trial.squareAvoid_ManikinTop.correct, 1);

list.accuracy_AvoidSquare.insertitem(trial.squareAvoid_ManikinTop.correct, 1);

if (trial.squareAvoid_ManikinTop.correct) {

list.latencies_allcorrecttrials.insertitem(trial.squareAvoid_ManikinTop.latency, 1);

list.latencies_corrAvoidSquare.insertitem(trial.squareAvoid_ManikinTop.latency, 1);

}

}

]

/ branch = [if (trial.squareAvoid_ManikinTop.response == 0) trial.trop_lent]

/ branch = [if (trial.squareAvoid_ManikinTop.error) trial.error]

/ correctmessage = (manikin_top_moveup, 1000)

/ timeout = 8000

</trial>

*******square picture man bottom avoid: 8

<trial squareAvoid_ManikinBottom>

/ ontrialbegin = [values.square_pictureindex = list.square_pictureindex.nextvalue]

/ stimulustimes = [0=clearscreen, manikinbottom; 1000=square]

/ validresponse = (parameters.responsekey_up, parameters.responsekey_down)

/ correctresponse = (parameters.responsekey_down)

/ ontrialend = [

if (values.test == 1) {

list.accuracy_alltrials.insertitem(trial.squareAvoid_ManikinBottom.correct, 1);

list.accuracy_AvoidSquare.insertitem(trial.squareAvoid_ManikinBottom.correct, 1);

if (trial.squareAvoid_ManikinBottom.correct) {

list.latencies_allcorrecttrials.insertitem(trial.squareAvoid_ManikinBottom.latency, 1);

list.latencies_corrAvoidSquare.insertitem(trial.squareAvoid_ManikinBottom.latency, 1);

}

}

]

/ branch = [if (trial.squareAvoid_ManikinBottom.response == 0) trial.trop_lent]

/ branch = [if (trial.squareAvoid_ManikinBottom.error) trial.error]

/ correctmessage = (manikin_bottom_movedown, 1000)

/ timeout = 8000

</trial>

<trial fixation>

/stimulustimes = [0=clearscreen, fixation]

/trialduration = (expressions.fixduration)

/correctresponse = (noresponse)

</trial>

<trial reminder>

/stimulustimes = [0=clearscreen, reminder]

/ correctresponse = (57)

</trial>

*** 48 trials per condition. The Fixation command makes the fixation cross appear on the screen.

<block approach_circle>

/ onblockbegin = [values.test = 1;]

/ screencolor = white

/ preinstructions = (approach_circle)

/ trials = [1,3,5,7,9,11,13,15,17,19,21,23,25,27,29,31,33,35,37,39,41,43,45,47,49,51,53,55,57,59,61,63,65,67,69,71,73,75,77,79,81,83,85,87,89,91,93,95 = fixation;

2,4,6,8,10,12,14,16,18,20,22,24,26,28,30,32,34,36,38,40,42,44,46,48,50,52,54,56,58,60,62,64,66,68,70,72,74,76,78,80,82,84,86,88,90,92,94,96 = noreplace (circleApproach_ManikinTop,circleApproach_ManikinBottom, squareAvoid_ManikinTop, squareAvoid_ManikinBottom)]

</block>

<block avoid_circle>

/ onblockbegin = [values.test = 1;]

/ screencolor = white

/ preinstructions = (avoid_circle)

/ trials = [1,3,5,7,9,11,13,15,17,19,21,23,25,27,29,31,33,35,37,39,41,43,45,47,49,51,53,55,57,59,61,63,65,67,69,71,73,75,77,79,81,83,85,87,89,91,93,95 = fixation;

2,4,6,8,10,12,14,16,18,20,22,24,26,28,30,32,34,36,38,40,42,44,46,48,50,52,54,56,58,60,62,64,66,68,70,72,74,76,78,80,82,84,86,88,90,92,94,96 = noreplace (circleAvoid_ManikinTop,circleAvoid_ManikinBottom,squareApproach_ManikinTop, squareApproach_ManikinBottom)]

</block>

<block approach_ap>

/ onblockbegin = [values.test = 1;]

/ screencolor = white

/ preinstructions = (approach_ap)

/ trials = [1,3,5,7,9,11,13,15,17,19,21,23,25,27,29,31,33,35,37,39,41,43,45,47,49,51,53,55,57,59,61,63,65,67,69,71,73,75,77,79,81,83,85,87,89,91,93,95 = fixation;

2,4,6,8,10,12,14,16,18,20,22,24,26,28,30,32,34,36,38,40,42,44,46,48,50,52,54,56,58,60,62,64,66,68,70,72,74,76,78,80,82,84,86,88,90,92,94,96 = noreplace (ApApproach_ManikinTop,ApApproach_ManikinBottom,SedenAvoid_ManikinTop,SedenAvoid_ManikinBottom)]

</block>

<block avoid_ap>

/ onblockbegin = [values.test = 1;]

/ screencolor = white

/ preinstructions = (avoid_ap)

/ trials = [1,3,5,7,9,11,13,15,17,19,21,23,25,27,29,31,33,35,37,39,41,43,45,47,49,51,53,55,57,59,61,63,65,67,69,71,73,75,77,79,81,83,85,87,89,91,93,95 = fixation;

2,4,6,8,10,12,14,16,18,20,22,24,26,28,30,32,34,36,38,40,42,44,46,48,50,52,54,56,58,60,62,64,66,68,70,72,74,76,78,80,82,84,86,88,90,92,94,96 = noreplace (ApAvoid_ManikinTop,ApAvoid_ManikinBottom,SedenApproach_ManikinTop,SedenApproach_ManikinBottom)]

</block>

<block images>

/ trials = [1= instructionimages]

</block>

<block reminder>

/ trials = [1=reminder]

</block>

<block Consent>

/trials = [1 = Consent]

/ screencolor = (white)

</block>

*****************

Informed consent

*****************

<html Consent>

/items = ("Consent_Form_EN.html")

/position = (50%, 40%)

/size = (70%, 70%)

</html>

<trial Consent>

/inputdevice = mouse

/stimulusframes = [1 = Consent, agree, disagree]

/validresponse = (agree, disagree)

/monkeyresponse = ("agree")

/ correctresponse = (agree)

/ontrialend = [if (trial.Consent.response == "disagree") script.abort()]

</trial>

<text agree>

/items = ("Oui, ~nJe veux participer.")

/position = (25%, 85%)

/ fontstyle = ("Arial", 2%, false, false, false, false, 5, 1)

/ txcolor = (white)

/ txbgcolor = (black)

/ size = (20%, 10%)

/ vjustify = center

</text>

<text disagree>

/items = ("Non, ~nJe ne veux pas participer.")

/position = (75%, 85%)

/ fontstyle = ("Arial", 2%, false, false, false, false, 5, 1)

/ txcolor = (white)

/ txbgcolor = (black)

/ size = (20%, 10%)

/vjustify = center

</text>

**************

Dermographics

**************

*** participant ***

<textbox participant>

/ caption = "Veuillez entrer ci-dessous votre code participant organisé de la façon suivante :

Écrivez en lettres majuscules :

1. Les deux premières lettres du prénom de votre premier parent (e.g., DA).

2. Les deux premières lettres du prénom de votre second parent (e.g., NI).

3. Les deux derniers numéros ou lettres de votre code postal (e.g., 18).

4. La dernière lettre de votre nom de famille (e.g., R).

5. Les lettres F et R qui correspondant à la langue dans laquelle vous passez l’étude (français, e.g., FR)."

/ textboxsize = (80, 15)

/ mask = alphanumeric

/ required = true

/ fontstyle = ("Arial", 2.5%, false, false, false, false, 5, 1)

</textbox>

<surveypage participant>

/ caption = " "

/ fontstyle = ("Arial", 2.5%, true, false, false, false, 5, 1)

/ questions = [1=participant]

/ finishlabel = "Suivant"

/ showpagenumbers = false

/ nextlabel = "Suivant"

/ backlabel = " "

</surveypage>

<block participant>

/ trials = [1=participant]

</block>

*** Demographics ***

*Age

<textbox age>

/ caption = "Quel est votre âge (en années) ?"

/ textboxsize = (80, 15)

/ mask = alphanumeric

/ fontstyle = ("Arial", 2.5%, false, false, false, false, 5, 1)

/ subcaption = "Répondez uniquement avec des chiffres."

/ subcaptionfontstyle = ("Arial", 2.5%, false, true, false, false, 5, 1)

/ required = true

</textbox>

<radiobuttons sex>

/ caption = "Quel est votre sexe ?"

/ options = ("Mâle", "Femelle")

/ fontstyle = ("Arial", 2.5%, false, false, false, false, 5, 1)

/ required = true

</radiobuttons>

<radiobuttons gender>

/ caption = "Quel est votre identité de genre ?"

/ options = ("Femme", "Homme", "Non binaire", "Femme transgenre", "Homme transgenre", "Autre", "Je préfère ne pas répondre")

/ fontstyle = ("Arial", 2.5%, false, false, false, false, 5, 1)

/ required = true

</radiobuttons>

<textbox height>

/ caption = "Quelle est votre taille en cm (e.g., pour 1m79, veuillez indiquer 179)"

/ textboxsize = (80, 15)

/ mask = positiveinteger

/ fontstyle = ("Arial", 2.5%, false, false, false, false, 5, 1)

/ subcaption = "Veuillez répondre avec des chiffres uniquement."

/ subcaptionfontstyle = ("Arial", 2.5%, false, true, false, false, 5, 1)

/ required = true

</textbox>

<textbox weight>

/ caption = "Quel est votre poids (en kg) ? "

/ textboxsize = (80, 15)

/ mask = positiveinteger

/ fontstyle = ("Arial", 2.5%, false, false, false, false, 5, 1)

/ subcaption = "Veuillez répondre avec des chiffres uniquement."

/ subcaptionfontstyle = ("Arial", 2.5%, false, true, false, false, 5, 1)

/ required = true

</textbox>

<textbox country>

/ caption = "Quel est votre pays de résidence ?"

/ textboxsize = (80, 15)

/ mask = alphanumeric

/ fontstyle = ("Arial", 2.5%, false, false, false, false, 5, 1)

/ subcaptionfontstyle = ("Arial", 2.5%, false, true, false, false, 5, 1)

/ required = false

</textbox>

<checkboxes chronic>

/ caption = "Certaines de ces affections ci-dessous ont-elles été décelées chez vous par un médecin? Si oui, veuillez sélectionner ces affections. Si non, veuillez sélectionner 'Aucune'."

/ options = ("Maladie cardiaque, insuffisance coronaire, angine de poitrine ou infarctus du myocarde ou tout autre problème cardiaque, y compris l'insuffisance cardiaque",

"Accident vasculaire cérébral ou maladie cérébro-vasculaire, attaque cérébrale",

"Hypertension artérielle, tension élevée",

"Cholestérol, hypercholestérolémie",

"Diabète, glycémie élevée",

"Maladie pulmonaire chronique, telle que bronchite chonique ou emphysème",

"Asthme",

"Polyarthrites, y compris arthrose et rhumatismes",

"Ostéoporose",

"Cancer ou tumeur maligne, y compris leucémie ou lymphome, à l'exclusion des cancers de la peau à évolution bénigne",

"Ulcère gastrique ou duodénal",

"Maladie de Parkinson",

"Fracture de hanche ou du fémur",

"Maladie d’Alzheimer, démence, troubles de fonctionnement du cerveau, sénilité ou toute autre dégradation sérieuse de la mémoire",

"Autres troubles affectifs ou émotionnels, incluant l'anxiété, problèmes nerveux ou psychiatriques",

"Polyarthrite rhumatoïde",

"Maladie rénale chronique",

"Autres affections non mentionnées",

"Aucune")

/ fontstyle = ("Arial", 2.5%, false, false, false, false, 5, 1)

/ required = true

</checkboxes>

<surveypage demographics>

/ fontstyle = ("Arial", 25, true, false, false, false, 5, 1)

/ questions = [1=age; 2=sex; 3=gender; 4=height; 5=weight; 6=country; 7=chronic]

/ finishlabel = "Suivant"

/ showpagenumbers = false

/ nextlabel = "Suivant"

/ backlabel = " "

/ subcaption = ""

/ subcaptionfontstyle = ("Arial", 2.8%, false, false, false, false, 5, 1)

</surveypage>

<block demographics>

/ trials = [1=demographics]

</block>

***************

Questionnaires

***************

<block survey>

/ trials = [1=IPAQ]

</block>

*IPAQ

<surveypage IPAQ>

/ caption = "Nous nous intéressons aux différents types d’activités physiques que vous faites dans votre vie quotidienne. Les questions suivantes portent sur le temps que vous avez passé à être actif physiquement au cours des 7 derniers jours.

~nPrenez votre temps pour vous rappeler le plus précisément possible le NOMBRE DE JOURS et le TEMPS PAR JOUR qui vous sont demandées. La précision de vos réponses est importante.

~nLes questions concernent les activités physiques que vous faites au travail, dans votre maison ou votre jardin, pour vos déplacements, et pendant votre temps libre."

/ fontstyle = ("Arial", 2.5%, false, false, false, false, 5, 1)

/ questions = [1=vigorous_d; 2=vigorous_m; 3=moderate_d; 4=moderate_m; 5=sedentary_d; 6=sedentary_m; 7=intention; 8=attitude1; 9=attitude2; 10=pain]

/ finishlabel = "Suivant"

/ showpagenumbers = false

/ nextlabel = "Suivant"

/ backlabel = " "

/ subcaptionfontstyle = ("Arial", 2.8%, false, false, false, false, 5, 1)

</surveypage>

<textbox vigorous_d>

/ caption = "Pensez à toutes les ACTIVITES INTENSES que vous avez faites au cours des 7 DERNIERS JOURS.

Les activités physiques intenses font référence aux activités qui vous demandent un effort physique important et vous font respirer beaucoup plus difficilement que normalement.

Au cours des 7 derniers jours, combien y a-t-il eu de jours au cours desquels vous avez fait des activités physiques INTENSES comme porter des charges lourdes, bêcher, faire du vélo rapidement ou faire de la gym aérobic ?

Nombre de jours par semaine :"

/ textboxsize = (10, 5)

/ fontstyle = ("Arial", 2.5%, false, false, false, false, 5, 1)

/ mask = alphanumeric

/ required = true

</textbox>

<textbox vigorous_m>

/ caption = "Combien de temps avez-vous habituellement passé à faire des activités physiques INTENSES pendant l'UN DE CES JOURS ?

Nombre de minutes par jour :"

/ textboxsize = (10, 5)

/ fontstyle = ("Arial", 2.5%, false, false, false, false, 5, 1)

/ mask = alphanumeric

/ required = true

</textbox>

<textbox moderate_d>

/ caption = "Pensez à toutes les ACTIVITES MODEREES que vous avez faites au cours des 7 DERNIERS JOURS.

Les activités physiques modérées font référence aux activités qui vous demandent un effort physique modéré et vous font respirer un peu plus difficilement que normalement.

Au cours des 7 derniers jours, combien y a-t-il eu de jours au cours desquels vous avez fait des activités physiques MODEREES comme porter des charges légères, faire du vélo tranquillement, ou jouer au tennis en double ?

N'incluez pas la marche dans votre réponse car ce n'est pas une activité physique modérée.

Nombre de jours :"

/ textboxsize = (10, 5)

/ fontstyle = ("Arial", 2.5%, false, false, false, false, 5, 1)

/ mask = alphanumeric

/ required = true

</textbox>

<textbox moderate_m>

/ caption = "Combien de temps avez-vous habituellement passé à faire des activités physiques MODEREES pendant l'UN DE CES JOURS ?

Nombre de minutes par jour :"

/ textboxsize = (10, 5)

/ fontstyle = ("Arial", 2.5%, false, false, false, false, 5, 1)

/ mask = alphanumeric

/ required = true

</textbox>

<textbox sedentary_d>

/ caption = "Cette question porte sur le temps que vous avez passé ASSIS au cours des 7 DERNIERS JOURS durant votre temps libre. Incluez le temps passé au travail, à la maison, pendant les cours et pendant les loisirs.

Cela peut inclure le temps passé assis à un bureau, à rendre visite à des amis, à lire ou à s'asseoir ou s'allonger pour regarder la télévision.

Au cours des 7 derniers jours, combien de jours avez-vous passé du temps ASSIS ?

Nombre de jours :"

/ textboxsize = (10, 5)

/ fontstyle = ("Arial", 2.5%, false, false, false, false, 5, 1)

/ mask = alphanumeric

/ required = true

</textbox>

<textbox sedentary_m>

/ caption = "Combien de temps avez-vous habituellement été ASSIS L'UN DE CES JOURS ?

Nombre de minutes par jour :"

/ textboxsize = (10, 5)

/ fontstyle = ("Arial", 2.5%, false, false, false, false, 5, 1)

/ mask = alphanumeric

/ required = true

</textbox>

* Intention

<radiobuttons intention>

/ caption = "Dans quelle mesure êtes-vous d'accord avec l'affirmation suivante :

Au cours des 7 prochains jours,

j'ai l'intention de faire au moins 150 minutes d'activité physique d'intensité modérée ;

ou au moins 75 minutes d'activité physique d'intensité vigoureuse ;

ou une combinaison équivalente d'activité physique d'intensité modérée et vigoureuse."

/ options = ("1~nComplètement ~nen ~ndésaccord","2","3","4~nNi en désaccord ~nNi en accord","5","6","7~nComplètement~nd'accord")

/ optionvalues = ("1", "2", "3", "4", "5", "6", "7")

/ fontstyle = ("Arial", 2.5%, false, false, false, false, 5, 1)

/ orientation = horizontalequal

/ required = true

</radiobuttons>

* Explicit affective attitude

<radiobuttons attitude1>

/ caption = "Pour moi, faire de l'activité physique régulièrement ..."

/ options = ("1~nDésagréable","2~n~n","3~n~n","4~n~n","5~n~n","6~n~n","7~nAgréable")

/ optionvalues = ("1", "2", "3", "4", "5", "6", "7")

/ fontstyle = ("Arial", 2.5%, false, false, false, false, 5, 1)

/ orientation = horizontalequal

/ required = true

</radiobuttons>

<radiobuttons attitude2>

/ caption = "Pour moi, faire de l'activité physique régulièrement ..."

/ options = ("1~nDéplaisant","2~n~n","3~n~n","4~n~n","5~n~n","6~n~n","7~nPlaisant")

/ optionvalues = ("1", "2", "3", "4", "5", "6", "7")

/ fontstyle = ("Arial", 2.5%, false, false, false, false, 5, 1)

/ orientation = horizontalequal

/ required = true

</radiobuttons>

*************

Randomization

*************

<expt>

/subjects = (1 of 4)

/groupassignment = random

/ groups = (1 of 4)

/ blocks = [

1 = Consent;

2 = participant;

3 = demographics;

4 = approach_circle;

5 = reminder;

6 = images;

7 = approach_ap;

8 = reminder;

9 = images;

10 = avoid_ap;

11 = reminder;

12 = avoid_circle;

13 = survey

]

/ preinstructions = (intro)

/ postinstructions = (end)

/ onexptend = [values.completed = 1]

</expt>

<expt>

/subjects = (2 of 4)

/ groups = (2 of 4)

/groupassignment = random

/ blocks = [

1 = Consent;

2 = participant;

3 = demographics;

4 = avoid_circle;

5 = reminder;

6 = images;

7 = avoid_ap;

8 = reminder;

9 = images;

10 = approach_ap;

11 = reminder;

12 = approach_circle;

13 = survey

]

/ preinstructions = (intro)

/ postinstructions = (end)

/ onexptend = [values.completed = 1]

</expt>

<expt>

/subjects = (3 of 4)

/groupassignment = random

/ groups = (3 of 4)

/ blocks = [

1 = Consent;

2 = participant;

3 = demographics;

4 = avoid_circle;

5 = reminder;

6 = images;

7 = approach_ap;

8 = reminder;

9 = images;

10 = avoid_ap;

11 = reminder;

12 = approach_circle;

13 = survey

]

/ preinstructions = (intro)

/ postinstructions = (end)

/ onexptend = [values.completed = 1]

</expt>

<expt>

/subjects = (4 of 4)

/ groups = (4 of 4)

/groupassignment = random

/ blocks = [

1 = Consent;

2 = participant;

3 = demographics;

4 = approach_circle;

5 = reminder;

6 = images;

7 = avoid_ap;

8 = reminder;

9 = images;

10 = approach_ap;

11 = reminder;

12 = avoid_circle;

13 = survey

]

/ preinstructions = (intro)

/ postinstructions = (end)

/ onexptend = [values.completed = 1]

</expt>
