# Supplementary material for: Automatic approach-avoidance tendency toward physical activity, sedentary, and neutral stimuli as a function of age, explicit affective attitude, and intention to be active
Source: Peer Community J. Author manuscript; Available in PMC 2024 Dec 10. (PMC7617180; doi:10.24072/pcjournal.246)
Supplement: Data, code, and supplemental material [file EMS201322-supplement-Data__code__and_supplemental_material.zip › Boisgontier-Lab-Aging_Approach-Avoid_Physical-Activity-0a8d854/Materials/Inquisit code_EN.docx]

Code for the English version of the approach avoidance task on Inquisit 6 (Millisecond Software, Version 6.5.2, <https://www.millisecond.com>)

<usermanual>

LIFESPAN STUDY (ENGLISH VERSION)

</usermanual>

<parameters>

/ responsekey_up = 22

/ responsekey_down = 49

/ upkeylabel = "U"

/ downkeylabel = "N"

</parameters>

<instruct>

/ fontstyle = ("Arial", 3%, false, false, false, false, 5, 1)

/ txcolor = black

/ lastlabel = ("Press the space bar to continue")

/ nextlabel = ("Press the space bar to continue")

</instruct>

<defaults >

/canvasaspectratio = (4,3)

/ minimumversion = "6.5.0.0"

/ fontstyle = ("Arial", 3%, false, false, false, false, 5, 1)

/txbgcolor = white

/ txcolor = (0, 0, 0)

/ inputdevice = keyboard

</defaults>

********************

Output of raw data

********************

<data>

/ separatefiles = true

/ columns = (textbox.participant.response, build, computer.platform, date, time, subject, group, blockcode, blocknum, trialcode, latency, correct, trialnum, response, script.elapsedtime,

parameters.responsekey_up, parameters.responsekey_down, values.ap_pictureindex, values.seden_pictureindex, values.circle_pictureindex, values.square_pictureindex,textbox.age.response,textbox.height.response,textbox.weight.response,radiobuttons.sex.response,radiobuttons.gender.response,textbox.country.response,checkboxes.chronic.response,

textbox.vigorous_d.response,textbox.vigorous_m.response,textbox.moderate_d.response,textbox.moderate_m.response,textbox.sedentary_d.response,textbox.sedentary_m.response,radiobuttons.intention.response,radiobuttons.attitude1.response,radiobuttons.attitude2.response,

expressions.propcorr_ApApproach, expressions.meanRT_ApApproach,

expressions.propcorr_ApAvoid, expressions.meanRT_ApAvoid,

expressions.propcorr_SedenApproach, expressions.meanRT_SedenApproach,

expressions.propcorr_SedenAvoid, expressions.meanRT_SedenAvoid,

expressions.propcorr_circleApproach, expressions.meanRT_circleApproach,

expressions.propcorr_circleAvoid, expressions.meanRT_circleAvoid,

expressions.propcorr_squareApproach, expressions.meanRT_squareApproach,

expressions.propcorr_squareAvoid, expressions.meanRT_squareAvoid)

</data>

************************

Approach-Avoidance Task

************************

<values>

/ completed = 0

/ ap_pictureindex = 0

/ seden_pictureindex = 0

/ circle_pictureindex = 0

/ square_pictureindex = 0

/ nextpicture = 0

/ selectap = 0

/ selectseden = 0

/ stimulus = ""

/ test = 0

</values>

*****

<expressions >

/ propcorr_total = list.accuracy_alltrials.mean

/ meanRT_total = list.latencies_allcorrecttrials.mean

/ propcorr_ApApproach = list.accuracy_ApproachAp.mean

/ meanRT_ApApproach = list.latencies_corrApproachAp.mean

/ propcorr_ApAvoid = list.accuracy_AvoidAp.mean

/ meanRT_ApAvoid = list.latencies_corrAvoidAp.mean

/ propcorr_SedenApproach = list.accuracy_ApproachSeden.mean

/ meanRT_SedenApproach = list.latencies_corrApproachSeden.mean

/ propcorr_SedenAvoid = list.accuracy_AvoidSeden.mean

/ meanRT_SedenAvoid = list.latencies_corrAvoidSeden.mean

/ propcorr_circleApproach = list.accuracy_ApproachCircle.mean

/ meanRT_circleApproach = list.latencies_corrApproachCircle.mean

/ propcorr_circleAvoid = list.accuracy_AvoidCircle.mean

/ meanRT_circleAvoid = list.latencies_corrAvoidCircle.mean

/ propcorr_squareApproach = list.accuracy_ApproachSquare.mean

/ meanRT_squareApproach = list.latencies_corrApproachSquare.mean

/ propcorr_squareAvoid = list.accuracy_AvoidSquare.mean

/ meanRT_squareAvoid = list.latencies_corrAvoidSquare.mean

</expressions>

<expressions>

/fixduration = rand(500,750)

</expressions>

<picture ap>

/ items = appics

/ position = (50%,50%)

/ erase = false

</picture>

<picture seden>

/ items = sedenpics

/ position = (50%,50%)

/ erase = false

</picture>

<picture circle>

/ items = circlepics

/ position = (50%,50%)

/ size = (20%, 20%)

/ erase = false

</picture>

<picture square>

/ items = squarepics

/ position = (50%,50%)

/ size = (20%, 20%)

/ erase = false

</picture>

*********

<page intro>

^Thank you for participating in this online study that is divided into 3 parts:

^^1- Informed consent and demographic questions.

^^2- The "Manikin task" in 4 different conditions.

^^3- Questionnaires.

^^You need to answer all the questions on the page to be able to move to the next page by pressing the "Next" button located at the bottom of the page.

^^Please press the space bar on your keyboard when you are ready to start.

</page>

<page end>

^^Thank you for your participation :)

</page>

***Manikin instructions

<page approach_circle>

^^Please locate the"<%parameters.upkeylabel%>" and "<%parameters.downkeylabel%>" keys on your keyboard. These 2 keys will be useful here.

^^In the upcoming task, a manikin that represents YOU will appear either at the top of the screen, or at the bottom.

^^Then, an image will appear in the center of the screen.

^^Your task is to move the manikin as quickly as possible according to what the image depicts.

^^ - If the image depicts CIRCLES and OVALS, you should move the manikin TOWARDS that circle (APPROACH CIRCLE).

^^ - If the image depicts SQUARES and RECTANGLES, you should move the manikin AWAY from that square (AVOID SQUARE).

^^With your index fingers, press the keyboard key "<%parameters.upkeylabel%>" to move the manikin up and the key "<%parameters.downkeylabel%>" to move it down.

^^Please press the SPACE BAR to start the session.

</page>

<page approach_ap>

^^Please locate the"<%parameters.upkeylabel%>" and "<%parameters.downkeylabel%>" keys on your keyboard. These 2 keys will be useful here.

^^In the upcoming task, a manikin that represents YOU will appear either at the top of the screen, or at the bottom.

^^Then, an image will appear in the center of the screen.

^^Your task is to move the manikin as quickly as possible according to what the image depicts.

^^ - If the image depicts a PHYSICAL ACTIVITY, you should move the manikin TOWARDS that image (APPROACH PHYSICAL ACTIVITY).

^^ - If the image depicts a SEDENTARY BEHAVIOR, you should move the manikin AWAY from that image (AVOID SEDENTARY BEHAVIOR).

^^With your index fingers, press the keyboard key "<%parameters.upkeylabel%>" to move the manikin up and the key "<%parameters.downkeylabel%>" to move it down.

^^Please press the SPACE BAR to start the session.

</page>

<page avoid_circle>

^^Please locate the"<%parameters.upkeylabel%>" and "<%parameters.downkeylabel%>" keys on your keyboard. These 2 keys will be useful here.

^^In the upcoming task, a manikin that represents YOU will appear either at the top of the screen, or at the bottom.

^^Then, an image will appear in the center of the screen.

^^Your task is to move the manikin as quickly as possible according to what the image depicts.

^^ - If the image depicts CIRCLES and OVALS, you should move the manikin AWAY from that circle (AVOID CIRCLE).

^^ - If the image depicts SQUARES and RECTANGLES, you should move the manikin TOWARDS that square (APPROACH SQUARE).

^^With your index fingers, press the keyboard key "<%parameters.upkeylabel%>" to move the manikin up and the key "<%parameters.downkeylabel%>" to move it down.

^^Please press the SPACE BAR to start the session.

</page>

<page avoid_ap>

^^Please locate the"<%parameters.upkeylabel%>" and "<%parameters.downkeylabel%>" keys on your keyboard. These 2 keys will be useful here.

^^In the upcoming task, a manikin that represents YOU will appear either at the top of the screen, or at the bottom.

^^Then, an image will appear in the center of the screen.

^^Your task is to move the manikin as quickly as possible according to what the image depicts.

^^ - If the image depicts a PHYSICAL ACTIVITY, you should move the manikin AWAY from that image (AVOID PHYSICAL ACTIVITY).

^^ - If the image depicts a SEDENTARY BEHAVIOR, you should move the manikin TOWARDS that image (APPROACH SEDENTARY BEHAVIOR).

^^With your index fingers, press the keyboard key "<%parameters.upkeylabel%>" to move the manikin up and the key "<%parameters.downkeylabel%>" to move it down.

^^Please press the SPACE BAR to start the session.

</page>

........

<text fixation>

/ items = ("+")

/ fontstyle = ("Arial", 5.00%, true)

</text>

<text reminder>

/ items = ("Congratulations on completing this part of the study.

Please read the following message carefully as the instructions change.

~nPlease press the space bar to continue")

/ fontstyle = ("Arial", 3.00%, true)

</text>

<picture manikintop>

/ items = ("manikin_2.jpg")

/ position = (50%, 20%)

</picture>

<picture manikin_top_moveup>

/ items = ("manikin_2.jpg")

/ position = (50%, 10%)

</picture>

<picture manikin_top_movedown>

/ items = ("manikin_2.jpg")

/ position = (50%, 30%)

</picture>

<picture manikinbottom>

/ items = ("manikin_2.jpg")

/ position = (50%, 80%)

</picture>

<picture manikin_bottom_moveup>

/ items = ("manikin_2.jpg")

/ position = (50%, 70%)

</picture>

<picture manikin_bottom_movedown>

/ items = ("manikin_2.jpg")

/ position = (50%, 90%)

</picture>

<text error>

/ fontstyle = ("arial", 22pt, true)

/ items = ("ERROR")

/ position = (50%,50%)

/ txcolor = (red)

/ erase = false

</text>

<text trop_lent>

/ fontstyle = ("arial", 22pt, true)

/ items = ("TOO SLOW")

/ position = (50%,50%)

/ txcolor = (red)

/ erase = false

</text>

<picture instructionimages>

/ items = ("instructionimages.png")

/ position = (50%,50%)

/ size = (80%, 80%)

</picture>

Note: the following 4 lists help determine which pictures are presented during the experimental trials

<list ap_pictureindex>

/ items = (1, 2, 3, 4, 5, 6)

/ selectionrate = trial

/ selectionmode = random

</list>

<list seden_pictureindex>

/ items = (7, 8, 9, 10, 11, 12)

/ selectionrate = trial

/ selectionmode = random

</list>

<list circle_pictureindex>

/ items = (13,14,15,16,17,18)

/ selectionrate = trial

/ selectionmode = random

</list>

<list square_pictureindex>

/ items = (19,20,21,22,23,24)

/ selectionrate = trial

/ selectionmode = random

</list>

*************************************************

Data Lists: used for descriptive statistics

store latencies/accuracy data

fill up during runtime

*************************************************

<list accuracy_alltrials>

</list>

<list latencies_allcorrecttrials>

</list>

<list accuracy_ApproachAp>

</list>

<list latencies_corrApproachAp>

</list>

<list accuracy_AvoidAp>

</list>

<list latencies_corrAvoidAp>

</list>

<list accuracy_ApproachSeden>

</list>

<list latencies_corrApproachSeden>

</list>

<list accuracy_AvoidSeden>

</list>

<list latencies_corrAvoidSeden>

</list>

<list accuracy_ApproachCircle>

</list>

<list latencies_corrApproachCircle>

</list>

<list accuracy_AvoidCircle>

</list>

<list latencies_corrAvoidCircle>

</list>

<list accuracy_ApproachSquare>

</list>

<list latencies_corrApproachSquare>

</list>

<list accuracy_AvoidSquare>

</list>

<list latencies_corrAvoidSquare>

</list>

............

<item appics>

/ 1 = "AP-COUR.jpg"

/ 2 = "AP-ESCAL.jpg"

/ 3 = "AP-FOOT.jpg"

/ 4 = "AP-NAT.jpg"

/ 5 = "AP-RANDO.jpg"

/ 6 = "AP-VEL.jpg"

</item>

<item sedenpics>

/ 7 = "SED-CANAP.jpg"

/ 8 = "SED-HAMAC.jpg"

/ 9 = "SED-JVID.jpg"

/ 10 = "SED-LECT.jpg"

/ 11 = "SED-ORDI.jpg"

/ 12 = "SED-TV.jpg"

</item>

<item circlepics>

/ 13 = "AP-NATr.jpg"

/ 14 = "AP-RANDOr.jpg"

/ 15 = "AP-VELr.jpg"

/ 16 = "SED-CANAPr.jpg"

/ 17 = "SED-HAMACr.jpg"

/ 18 = "SED-LECTr.jpg"

</item>

<item squarepics>

/ 19 = "AP-NATc.jpg"

/ 20 = "AP-RANDOc.jpg"

/ 21 = "AP-VELc.jpg"

/ 22 = "SED-CANAPc.jpg"

/ 23 = "SED-HAMACc.jpg"

/ 24 = "SED-LECTc.jpg"

</item>

........

........

<trial error>

/ trialcode = "error"

/ stimulustimes = [0= error]

/ trialduration = (800)

/ responsemode = noresponse

</trial>

<trial trop_lent>

/ trialcode = "too_slow"

/ stimulustimes = [0= trop_lent]

/ trialduration = (800)

/ responsemode = noresponse

</trial>

<trial instructionimages>

/trialcode = "instructionimages"

/ stimulustimes = [0=instructionimages]

/ trialduration = 7000

</trial>

*******physical activity picture man top approach: 1

<trial ApApproach_ManikinTop>

/ ontrialbegin = [values.ap_pictureindex = list.ap_pictureindex.nextvalue]

/ stimulustimes = [0 = clearscreen, manikintop; 1000=ap]

/ validresponse = (parameters.responsekey_up, parameters.responsekey_down)

/ correctresponse = (parameters.responsekey_down)

/ ontrialend = [

if (values.test == 1) {

list.accuracy_alltrials.insertitem(trial.ApApproach_ManikinTop.correct, 1);

list.accuracy_ApproachAp.insertitem(trial.ApApproach_ManikinTop.correct, 1);

if (trial.ApApproach_ManikinTop.correct) {

list.latencies_allcorrecttrials.insertitem(trial.ApApproach_ManikinTop.latency, 1);

list.latencies_corrApproachAp.insertitem(trial.ApApproach_ManikinTop.latency, 1);

}

}

]

/ correctmessage = (manikin_top_movedown, 1000)

/ branch = [if (trial.ApApproach_ManikinTop.response == 0) trial.trop_lent]

/ branch = [if (trial.ApApproach_ManikinTop.error) trial.error]

/ timeout = 8000

</trial>

*******physical activity picture man bottom approach: 2

<trial ApApproach_ManikinBottom>

/ ontrialbegin = [values.ap_pictureindex = list.ap_pictureindex.nextvalue]

/ stimulustimes = [0 = clearscreen, manikinbottom; 1000=ap]

/ validresponse = (parameters.responsekey_up, parameters.responsekey_down)

/ correctresponse = (parameters.responsekey_up)

/ ontrialend = [

if (values.test == 1) {

list.accuracy_alltrials.insertitem(trial.ApApproach_ManikinBottom.correct, 1);

list.accuracy_ApproachAp.insertitem(trial.ApApproach_ManikinBottom.correct, 1);

if (trial.ApApproach_ManikinBottom.correct) {

list.latencies_allcorrecttrials.insertitem(trial.ApApproach_ManikinBottom.latency, 1);

list.latencies_corrApproachAp.insertitem(trial.ApApproach_ManikinBottom.latency, 1);

}

}

]

/ branch = [if (trial.ApApproach_ManikinBottom.response == 0) trial.trop_lent]

/ branch = [if (trial.ApApproach_ManikinBottom.error) trial.error]

/ correctmessage = (manikin_bottom_moveup, 1000)

/ timeout = 8000

</trial>

*******physical activity picture man top avoid: 3

<trial ApAvoid_ManikinTop>

/ ontrialbegin = [values.ap_pictureindex = list.ap_pictureindex.nextvalue]

/ stimulustimes = [0 = clearscreen, manikintop; 1000=ap]

/ validresponse = (parameters.responsekey_up, parameters.responsekey_down)

/ correctresponse = (parameters.responsekey_up)

/ ontrialend = [

if (values.test == 1) {

list.accuracy_alltrials.insertitem(trial.ApAvoid_ManikinTop.correct, 1);

list.accuracy_AvoidAp.insertitem(trial.ApAvoid_ManikinTop.correct, 1);

if (trial.ApAvoid_ManikinTop.correct) {

list.latencies_allcorrecttrials.insertitem(trial.ApAvoid_ManikinTop.latency, 1);

list.latencies_corrAvoidAp.insertitem(trial.ApAvoid_ManikinTop.latency, 1);

}

}

]

/ branch = [if (trial.ApAvoid_ManikinTop.response == 0) trial.trop_lent]

/ branch = [if (trial.ApAvoid_ManikinTop.error) trial.error]

/ correctmessage = (manikin_top_moveup, 1000)

/ timeout = 8000

</trial>

*******physical activity picture man bottom avoid: 4

<trial ApAvoid_ManikinBottom>

/ ontrialbegin = [values.ap_pictureindex = list.ap_pictureindex.nextvalue]

/ stimulustimes = [0 = clearscreen, manikinbottom; 1000=ap]

/ validresponse = (parameters.responsekey_up, parameters.responsekey_down)

/ correctresponse = (parameters.responsekey_down)

/ ontrialend = [

if (values.test == 1) {

list.accuracy_alltrials.insertitem(trial.ApAvoid_ManikinBottom.correct, 1);

list.accuracy_AvoidAp.insertitem(trial.ApAvoid_ManikinBottom.correct, 1);

if (trial.ApAvoid_ManikinBottom.correct) {

list.latencies_allcorrecttrials.insertitem(trial.ApAvoid_ManikinBottom.latency, 1);

list.latencies_corrAvoidAp.insertitem(trial.ApAvoid_ManikinBottom.latency, 1);

}

}

]

/ branch = [if (trial.ApAvoid_ManikinBottom.response == 0) trial.trop_lent]

/ branch = [if (trial.ApAvoid_ManikinBottom.error) trial.error]

/ correctmessage = (manikin_bottom_movedown, 1000)

/ timeout = 8000

</trial>

*******sedentary picture man top approach: 5

<trial SedenApproach_ManikinTop>

/ ontrialbegin = [values.seden_pictureindex = list.seden_pictureindex.nextvalue]

/ stimulustimes = [0 = clearscreen, manikintop; 1000=seden]

/ validresponse = (parameters.responsekey_up, parameters.responsekey_down)

/ correctresponse = (parameters.responsekey_down)

/ ontrialend = [

if (values.test == 1) {

list.accuracy_alltrials.insertitem(trial.SedenApproach_ManikinTop.correct, 1);

list.accuracy_ApproachSeden.insertitem(trial.SedenApproach_ManikinTop.correct, 1);

if (trial.SedenApproach_ManikinTop.correct) {

list.latencies_allcorrecttrials.insertitem(trial.SedenApproach_ManikinTop.latency, 1);

list.latencies_corrApproachSeden.insertitem(trial.SedenApproach_ManikinTop.latency, 1);

}

}

]

/ branch = [if (trial.SedenApproach_ManikinTop.response == 0) trial.trop_lent]

/ branch = [if (trial.SedenApproach_ManikinTop.error) trial.error]

/ correctmessage = (manikin_top_movedown, 1000)

/ timeout = 8000

</trial>

*******sedentary picture man bottom approach: 6

<trial SedenApproach_ManikinBottom>

/ ontrialbegin = [values.seden_pictureindex = list.seden_pictureindex.nextvalue]

/ stimulustimes = [0=clearscreen, manikinbottom; 1000=seden]

/ validresponse = (parameters.responsekey_up, parameters.responsekey_down)

/ correctresponse = (parameters.responsekey_up)

/ ontrialend = [

if (values.test == 1) {

list.accuracy_alltrials.insertitem(trial.SedenApproach_ManikinBottom.correct, 1);

list.accuracy_ApproachSeden.insertitem(trial.SedenApproach_ManikinBottom.correct, 1);

if (trial.SedenApproach_ManikinBottom.correct) {

list.latencies_allcorrecttrials.insertitem(trial.SedenApproach_ManikinBottom.latency, 1);

list.latencies_corrApproachSeden.insertitem(trial.SedenApproach_ManikinBottom.latency, 1);

}

}

]

/ branch = [if (trial.SedenApproach_ManikinBottom.response == 0) trial.trop_lent]

/ branch = [if (trial.SedenApproach_ManikinBottom.error) trial.error]

/ correctmessage = (manikin_bottom_moveup, 1000)

/ timeout = 8000

</trial>

*******sedentaryt picture man top avoid: 7

<trial SedenAvoid_ManikinTop>

/ ontrialbegin = [values.seden_pictureindex = list.seden_pictureindex.nextvalue]

/ stimulustimes = [0 = clearscreen, manikintop; 1000=seden]

/ validresponse = (parameters.responsekey_up, parameters.responsekey_down)

/ correctresponse = (parameters.responsekey_up)

/ ontrialend = [

if (values.test == 1) {

list.accuracy_alltrials.insertitem(trial.SedenAvoid_ManikinTop.correct, 1);

list.accuracy_AvoidSeden.insertitem(trial.SedenAvoid_ManikinTop.correct, 1);

if (trial.SedenAvoid_ManikinTop.correct) {

list.latencies_allcorrecttrials.insertitem(trial.SedenAvoid_ManikinTop.latency, 1);

list.latencies_corrAvoidSeden.insertitem(trial.SedenAvoid_ManikinTop.latency, 1);

}

}

]

/ branch = [if (trial.SedenAvoid_ManikinTop.response == 0) trial.trop_lent]

/ branch = [if (trial.SedenAvoid_ManikinTop.error) trial.error]

/ correctmessage = (manikin_top_moveup, 1000)

/ timeout = 8000

</trial>

*******sedentary picture man bottom avoid: 8

<trial SedenAvoid_ManikinBottom>

/ ontrialbegin = [values.seden_pictureindex = list.seden_pictureindex.nextvalue]

/ stimulustimes = [0=clearscreen, manikinbottom; 1000=seden]

/ validresponse = (parameters.responsekey_up, parameters.responsekey_down)

/ correctresponse = (parameters.responsekey_down)

/ ontrialend = [

if (values.test == 1) {

list.accuracy_alltrials.insertitem(trial.SedenAvoid_ManikinBottom.correct, 1);

list.accuracy_AvoidSeden.insertitem(trial.SedenAvoid_ManikinBottom.correct, 1);

if (trial.SedenAvoid_ManikinBottom.correct) {

list.latencies_allcorrecttrials.insertitem(trial.SedenAvoid_ManikinBottom.latency, 1);

list.latencies_corrAvoidSeden.insertitem(trial.SedenAvoid_ManikinBottom.latency, 1);

}

}

]

/ branch = [if (trial.SedenAvoid_ManikinBottom.response == 0) trial.trop_lent]

/ branch = [if (trial.SedenAvoid_ManikinBottom.error) trial.error]

/ correctmessage = (manikin_bottom_movedown, 1000)

/ timeout = 8000

</trial>

*******circle picture man top approach: 1

<trial circleApproach_ManikinTop>

/ ontrialbegin = [values.circle_pictureindex = list.circle_pictureindex.nextvalue]

/ stimulustimes = [0 = clearscreen, manikintop; 1000=circle]

/ validresponse = (parameters.responsekey_up, parameters.responsekey_down)

/ correctresponse = (parameters.responsekey_down)

/ ontrialend = [

if (values.test == 1) {

list.accuracy_alltrials.insertitem(trial.circleApproach_ManikinTop.correct, 1);

list.accuracy_ApproachCircle.insertitem(trial.circleApproach_ManikinTop.correct, 1);

if (trial.circleApproach_ManikinTop.correct) {

list.latencies_allcorrecttrials.insertitem(trial.circleApproach_ManikinTop.latency, 1);

list.latencies_corrApproachCircle.insertitem(trial.circleApproach_ManikinTop.latency, 1);

}

}

]

/ correctmessage = (manikin_top_movedown, 1000)

/ branch = [if (trial.circleApproach_ManikinTop.response == 0) trial.trop_lent]

/ branch = [if (trial.circleApproach_ManikinTop.error) trial.error]

/ timeout = 8000

</trial>

*******circle picture man bottom approach: 2

<trial circleApproach_ManikinBottom>

/ ontrialbegin = [values.circle_pictureindex = list.circle_pictureindex.nextvalue]

/ stimulustimes = [0 = clearscreen, manikinbottom; 1000=circle]

/ validresponse = (parameters.responsekey_up, parameters.responsekey_down)

/ correctresponse = (parameters.responsekey_up)

/ ontrialend = [

if (values.test == 1) {

list.accuracy_alltrials.insertitem(trial.circleApproach_ManikinBottom.correct, 1);

list.accuracy_ApproachCircle.insertitem(trial.circleApproach_ManikinBottom.correct, 1);

if (trial.circleApproach_ManikinBottom.correct) {

list.latencies_allcorrecttrials.insertitem(trial.circleApproach_ManikinBottom.latency, 1);

list.latencies_corrApproachCircle.insertitem(trial.circleApproach_ManikinBottom.latency, 1);

}

}

]

/ branch = [if (trial.circleApproach_ManikinBottom.response == 0) trial.trop_lent]

/ branch = [if (trial.circleApproach_ManikinBottom.error) trial.error]

/ correctmessage = (manikin_bottom_moveup, 1000)

/ timeout = 8000

</trial>

*******circle picture man top avoid: 3

<trial circleAvoid_ManikinTop>

/ ontrialbegin = [values.circle_pictureindex = list.circle_pictureindex.nextvalue]

/ stimulustimes = [0 = clearscreen, manikintop; 1000=circle]

/ validresponse = (parameters.responsekey_up, parameters.responsekey_down)

/ correctresponse = (parameters.responsekey_up)

/ ontrialend = [

if (values.test == 1) {

list.accuracy_alltrials.insertitem(trial.circleAvoid_ManikinTop.correct, 1);

list.accuracy_AvoidCircle.insertitem(trial.circleAvoid_ManikinTop.correct, 1);

if (trial.circleAvoid_ManikinTop.correct) {

list.latencies_allcorrecttrials.insertitem(trial.circleAvoid_ManikinTop.latency, 1);

list.latencies_corrAvoidCircle.insertitem(trial.circleAvoid_ManikinTop.latency, 1);

}

}

]

/ branch = [if (trial.circleAvoid_ManikinTop.response == 0) trial.trop_lent]

/ branch = [if (trial.circleAvoid_ManikinTop.error) trial.error]

/ correctmessage = (manikin_top_moveup, 1000)

/ timeout = 8000

</trial>

*******circle picture man bottom avoid: 4

<trial circleAvoid_ManikinBottom>

/ ontrialbegin = [values.circle_pictureindex = list.circle_pictureindex.nextvalue]

/ stimulustimes = [0 = clearscreen, manikinbottom; 1000=circle]

/ validresponse = (parameters.responsekey_up, parameters.responsekey_down)

/ correctresponse = (parameters.responsekey_down)

/ ontrialend = [

if (values.test == 1) {

list.accuracy_alltrials.insertitem(trial.circleAvoid_ManikinBottom.correct, 1);

list.accuracy_AvoidCircle.insertitem(trial.circleAvoid_ManikinBottom.correct, 1);

if (trial.circleAvoid_ManikinBottom.correct) {

list.latencies_allcorrecttrials.insertitem(trial.circleAvoid_ManikinBottom.latency, 1);

list.latencies_corrAvoidCircle.insertitem(trial.circleAvoid_ManikinBottom.latency, 1);

}

}

]

/ branch = [if (trial.circleAvoid_ManikinBottom.response == 0) trial.trop_lent]

/ branch = [if (trial.circleAvoid_ManikinBottom.error) trial.error]

/ correctmessage = (manikin_bottom_movedown, 1000)

/ timeout = 8000

</trial>

*******square picture man top approach: 5

<trial squareApproach_ManikinTop>

/ ontrialbegin = [values.square_pictureindex = list.square_pictureindex.nextvalue]

/ stimulustimes = [0 = clearscreen, manikintop; 1000=square]

/ validresponse = (parameters.responsekey_up, parameters.responsekey_down)

/ correctresponse = (parameters.responsekey_down)

/ ontrialend = [

if (values.test == 1) {

list.accuracy_alltrials.insertitem(trial.squareApproach_ManikinTop.correct, 1);

list.accuracy_ApproachSquare.insertitem(trial.squareApproach_ManikinTop.correct, 1);

if (trial.squareApproach_ManikinTop.correct) {

list.latencies_allcorrecttrials.insertitem(trial.squareApproach_ManikinTop.latency, 1);

list.latencies_corrApproachSquare.insertitem(trial.squareApproach_ManikinTop.latency, 1);

}

}

]

/ branch = [if (trial.squareApproach_ManikinTop.response == 0) trial.trop_lent]

/ branch = [if (trial.squareApproach_ManikinTop.error) trial.error]

/ correctmessage = (manikin_top_movedown, 1000)

/ timeout = 8000

</trial>

*******square picture man bottom approach: 6

<trial squareApproach_ManikinBottom>

/ ontrialbegin = [values.square_pictureindex = list.square_pictureindex.nextvalue]

/ stimulustimes = [0=clearscreen, manikinbottom; 1000=square]

/ validresponse = (parameters.responsekey_up, parameters.responsekey_down)

/ correctresponse = (parameters.responsekey_up)

/ ontrialend = [

if (values.test == 1) {

list.accuracy_alltrials.insertitem(trial.squareApproach_ManikinBottom.correct, 1);

list.accuracy_ApproachSquare.insertitem(trial.squareApproach_ManikinBottom.correct, 1);

if (trial.squareApproach_ManikinBottom.correct) {

list.latencies_allcorrecttrials.insertitem(trial.squareApproach_ManikinBottom.latency, 1);

list.latencies_corrApproachSquare.insertitem(trial.squareApproach_ManikinBottom.latency, 1);

}

}

]

/ branch = [if (trial.squareApproach_ManikinBottom.response == 0) trial.trop_lent]

/ branch = [if (trial.squareApproach_ManikinBottom.error) trial.error]

/ correctmessage = (manikin_bottom_moveup, 1000)

/ timeout = 8000

</trial>

*******square picture man top avoid: 7

<trial squareAvoid_ManikinTop>

/ ontrialbegin = [values.square_pictureindex = list.square_pictureindex.nextvalue]

/ stimulustimes = [0 = clearscreen, manikintop; 1000=square]

/ validresponse = (parameters.responsekey_up, parameters.responsekey_down)

/ correctresponse = (parameters.responsekey_up)

/ ontrialend = [

if (values.test == 1) {

list.accuracy_alltrials.insertitem(trial.squareAvoid_ManikinTop.correct, 1);

list.accuracy_AvoidSquare.insertitem(trial.squareAvoid_ManikinTop.correct, 1);

if (trial.squareAvoid_ManikinTop.correct) {

list.latencies_allcorrecttrials.insertitem(trial.squareAvoid_ManikinTop.latency, 1);

list.latencies_corrAvoidSquare.insertitem(trial.squareAvoid_ManikinTop.latency, 1);

}

}

]

/ branch = [if (trial.squareAvoid_ManikinTop.response == 0) trial.trop_lent]

/ branch = [if (trial.squareAvoid_ManikinTop.error) trial.error]

/ correctmessage = (manikin_top_moveup, 1000)

/ timeout = 8000

</trial>

*******square picture man bottom avoid: 8

<trial squareAvoid_ManikinBottom>

/ ontrialbegin = [values.square_pictureindex = list.square_pictureindex.nextvalue]

/ stimulustimes = [0=clearscreen, manikinbottom; 1000=square]

/ validresponse = (parameters.responsekey_up, parameters.responsekey_down)

/ correctresponse = (parameters.responsekey_down)

/ ontrialend = [

if (values.test == 1) {

list.accuracy_alltrials.insertitem(trial.squareAvoid_ManikinBottom.correct, 1);

list.accuracy_AvoidSquare.insertitem(trial.squareAvoid_ManikinBottom.correct, 1);

if (trial.squareAvoid_ManikinBottom.correct) {

list.latencies_allcorrecttrials.insertitem(trial.squareAvoid_ManikinBottom.latency, 1);

list.latencies_corrAvoidSquare.insertitem(trial.squareAvoid_ManikinBottom.latency, 1);

}

}

]

/ branch = [if (trial.squareAvoid_ManikinBottom.response == 0) trial.trop_lent]

/ branch = [if (trial.squareAvoid_ManikinBottom.error) trial.error]

/ correctmessage = (manikin_bottom_movedown, 1000)

/ timeout = 8000

</trial>

<trial fixation>

/stimulustimes = [0=clearscreen, fixation]

/trialduration = (expressions.fixduration)

/correctresponse = (noresponse)

</trial>

<trial reminder>

/stimulustimes = [0=clearscreen, reminder]

/ correctresponse = (57)

</trial>

*** 48 trials per condition. The Fixation command makes the fixation cross appear on the screen.

<block approach_circle>

/ onblockbegin = [values.test = 1;]

/ screencolor = white

/ preinstructions = (approach_circle)

/ trials = [1,3,5,7,9,11,13,15,17,19,21,23,25,27,29,31,33,35,37,39,41,43,45,47,49,51,53,55,57,59,61,63,65,67,69,71,73,75,77,79,81,83,85,87,89,91,93,95 = fixation;

2,4,6,8,10,12,14,16,18,20,22,24,26,28,30,32,34,36,38,40,42,44,46,48,50,52,54,56,58,60,62,64,66,68,70,72,74,76,78,80,82,84,86,88,90,92,94,96 = noreplace (circleApproach_ManikinTop,circleApproach_ManikinBottom, squareAvoid_ManikinTop, squareAvoid_ManikinBottom)]

</block>

<block avoid_circle>

/ onblockbegin = [values.test = 1;]

/ screencolor = white

/ preinstructions = (avoid_circle)

/ trials = [1,3,5,7,9,11,13,15,17,19,21,23,25,27,29,31,33,35,37,39,41,43,45,47,49,51,53,55,57,59,61,63,65,67,69,71,73,75,77,79,81,83,85,87,89,91,93,95 = fixation;

2,4,6,8,10,12,14,16,18,20,22,24,26,28,30,32,34,36,38,40,42,44,46,48,50,52,54,56,58,60,62,64,66,68,70,72,74,76,78,80,82,84,86,88,90,92,94,96 = noreplace (circleAvoid_ManikinTop,circleAvoid_ManikinBottom,squareApproach_ManikinTop, squareApproach_ManikinBottom)]

</block>

<block approach_ap>

/ onblockbegin = [values.test = 1;]

/ screencolor = white

/ preinstructions = (approach_ap)

/ trials = [1,3,5,7,9,11,13,15,17,19,21,23,25,27,29,31,33,35,37,39,41,43,45,47,49,51,53,55,57,59,61,63,65,67,69,71,73,75,77,79,81,83,85,87,89,91,93,95 = fixation;

2,4,6,8,10,12,14,16,18,20,22,24,26,28,30,32,34,36,38,40,42,44,46,48,50,52,54,56,58,60,62,64,66,68,70,72,74,76,78,80,82,84,86,88,90,92,94,96 = noreplace (ApApproach_ManikinTop,ApApproach_ManikinBottom,SedenAvoid_ManikinTop,SedenAvoid_ManikinBottom)]

</block>

<block avoid_ap>

/ onblockbegin = [values.test = 1;]

/ screencolor = white

/ preinstructions = (avoid_ap)

/ trials = [1,3,5,7,9,11,13,15,17,19,21,23,25,27,29,31,33,35,37,39,41,43,45,47,49,51,53,55,57,59,61,63,65,67,69,71,73,75,77,79,81,83,85,87,89,91,93,95 = fixation;

2,4,6,8,10,12,14,16,18,20,22,24,26,28,30,32,34,36,38,40,42,44,46,48,50,52,54,56,58,60,62,64,66,68,70,72,74,76,78,80,82,84,86,88,90,92,94,96 = noreplace (ApAvoid_ManikinTop,ApAvoid_ManikinBottom,SedenApproach_ManikinTop,SedenApproach_ManikinBottom)]

</block>

<block images>

/ trials = [1= instructionimages]

</block>

<block reminder>

/ trials = [1=reminder]

</block>

<block Consent>

/trials = [1 = Consent]

/ screencolor = (white)

</block>

*****************

Informed consent

*****************

<html Consent>

/items = ("Consent_Form_EN.html")

/position = (50%, 40%)

/size = (70%, 70%)

</html>

<trial Consent>

/inputdevice = mouse

/stimulusframes = [1 = Consent, agree, disagree]

/validresponse = (agree, disagree)

/monkeyresponse = ("agree")

/ correctresponse = (agree)

/ontrialend = [if (trial.Consent.response == "disagree") script.abort()]

</trial>

<text agree>

/items = ("Yes, ~nI want to participate.")

/position = (25%, 85%)

/ fontstyle = ("Arial", 2%, false, false, false, false, 5, 1)

/ txcolor = (white)

/ txbgcolor = (black)

/ size = (20%, 10%)

/ vjustify = center

</text>

<text disagree>

/items = ("No, ~nI do not want to participate.")

/position = (75%, 85%)

/ fontstyle = ("Arial", 2%, false, false, false, false, 5, 1)

/ txcolor = (white)

/ txbgcolor = (black)

/ size = (20%, 10%)

/vjustify = center

</text>

**************

Dermographics

**************

*** participant ***

<textbox participant>

/ caption = "Please enter your participant code below, organized as follows:

Please write in capital letters:

1. The first two letters of your first parent's first name (e.g., DA)

2. The first two letters of your second parent’s first name (e.g., NI)

3. The last two letters or numbers of your postal code (e.g.,K5)

4. The last letter of your last name (e.g., M)

5. The letters E and N corresponding to the language in which you are completing the study (English, e.g., EN)"

/ textboxsize = (80, 15)

/ mask = alphanumeric

/ required = true

/ fontstyle = ("Arial", 2.5%, false, false, false, false, 5, 1)

</textbox>

<surveypage participant>

/ caption = " "

/ fontstyle = ("Arial", 2.5%, true, false, false, false, 5, 1)

/ questions = [1=participant]

/ finishlabel = "Next"

/ showpagenumbers = false

/ nextlabel = "Next"

/ backlabel = " "

</surveypage>

<block participant>

/ trials = [1=participant]

</block>

*** Demographics ***

<textbox age>

/ caption = "What is your age (in years)?"

/ textboxsize = (80, 15)

/ mask = alphanumeric

/ fontstyle = ("Arial", 2.5%, false, false, false, false, 5, 1)

/ subcaption = "Please answer this question with only numbers."

/ subcaptionfontstyle = ("Arial", 2.5%, false, true, false, false, 5, 1)

/ required = true

</textbox>

<radiobuttons sex>

/ caption = "What is your sex?"

/ options = ("Male", "Female")

/ fontstyle = ("Arial", 2.5%, false, false, false, false, 5, 1)

/ required = true

</radiobuttons>

<radiobuttons gender>

/ caption = "What is your gender identity?"

/ options = ("Man", "Woman", "Non binary", "Transgender man", "Transgender woman", "Other", "I prefer not to answer")

/ fontstyle = ("Arial", 2.5%, false, false, false, false, 5, 1)

/ required = true

</radiobuttons>

<textbox height>

/ caption = "What is your height in cm (e.g., for 1m79, please indicate 179)"

/ textboxsize = (80, 15)

/ mask = positiveinteger

/ fontstyle = ("Arial", 2.5%, false, false, false, false, 5, 1)

/ subcaption = "Please answer with numbers only."

/ subcaptionfontstyle = ("Arial", 2.5%, false, true, false, false, 5, 1)

/ required = true

</textbox>

<textbox weight>

/ caption = "What is your weight in kg? If you need to convert your weight in kg from lb please do lb/2.2"

/ textboxsize = (80, 15)

/ mask = positiveinteger

/ fontstyle = ("Arial", 2.5%, false, false, false, false, 5, 1)

/ subcaption = "Please answer this question with numbers only."

/ subcaptionfontstyle = ("Arial", 2.5%, false, true, false, false, 5, 1)

/ required = true

</textbox>

<textbox country>

/ caption = "What is your country of residence?"

/ textboxsize = (80, 15)

/ mask = alphanumeric

/ fontstyle = ("Arial", 2.5%, false, false, false, false, 5, 1)

/ subcaptionfontstyle = ("Arial", 2.5%, false, true, false, false, 5, 1)

/ required = false

</textbox>

*Chronic health condition

<checkboxes chronic>

/ caption = "Has a doctor ever told you that you had any of the following conditions? If yes, please selected the conditions. If not, please select 'None'"

/ options = ("A heart attack including myocardial infarction or coronary thrombosis or any other heart problem including congestive heart failure",

"A stroke or cerebral vascular disease",

"High blood pressure or hypertension",

"High blood cholesterol",

"Diabetes or high blood sugar",

"Chronic lung disease such as chronic bronchitis or emphysema",

"Asthma",

"Arthritis, including osteoarthritis, or rheumatism",

"Osteoporosis",

"Cancer or malignant tumour, including leukaemia or lymphoma, but excluding minor skin cancers",

"Stomach or duodental ulcer, peptic ulcer",

"Parkinson disease",

"Hip fracture or femoral fracture",

"Alzheimer’s disease, dementia, organic brain syndrome, senility or any other serious memory impairment",

"Other affective or emotional disorders, including anxiety, nervous or psychiatric problems",

"Rheumatoid Arthritis",

"Chronic kidney disease",

"Other conditions, not yet mentioned",

"None")

/ fontstyle = ("Arial", 2.5%, false, false, false, false, 5, 1)

/ required = true

</checkboxes>

<surveypage demographics>

/ fontstyle = ("Arial", 25, true, false, false, false, 5, 1)

/ questions = [1=age; 2=sex; 3=gender; 4=height; 5=weight; 6=country; 7=chronic]

/ finishlabel = "Next"

/showpagenumbers = false

/ nextlabel = "Next"

/backlabel = " "

/ subcaption = ""

/ subcaptionfontstyle = ("Arial", 2.8%, false, false, false, false, 5, 1)

</surveypage>

<block demographics>

/ trials = [1=demographics]

</block>

***************

Questionnaires

***************

<block survey>

/ trials = [1=IPAQ]

</block>

*IPAQ

<surveypage IPAQ>

/ caption = "We are interested in finding out about the kinds of physical activities that people do as part of their everyday lives.

The questions will ask you about the amount of time you spent being physically active in the last 7 days.

Please answer each question even if you do not consider yourself to be an active person.

Please think about the the NUMBER OF DAYS and the TIME SPENT EACH OF THESE DAYS doing activities you do at work, as part of your house, and yard work,

to get from place to place, and in your spare time for recreation, exercise or sport."

/ fontstyle = ("Arial", 2.5%, false, false, false, false, 5, 1)

/ questions = [1=vigorous_d; 2=vigorous_m; 3=moderate_d; 4=moderate_m; 5=sedentary_d; 6=sedentary_m; 7=intention; 8=attitude1; 9=attitude2; 10=pain]

/ finishlabel = "Next"

/ showpagenumbers = false

/ nextlabel = "Next"

/ backlabel = " "

/ subcaptionfontstyle = ("Arial", 2.8%, false, false, false, false, 5, 1)

</surveypage>

<textbox vigorous_d>

/ caption = "Think about all the VIGOROUS activities that you did in the LAST 7 DAYS. Vigorous physical activities refer to activities that take hard physical effort and make you breathe much harder than normal.

During the last 7 days, on how many days did you do vigorous physical activities like heavy lifting, digging, aerobics, or fast bicycling?

Number of days per week:"

/ textboxsize = (10, 5)

/ fontstyle = ("Arial", 2.5%, false, false, false, false, 5, 1)

/ mask = alphanumeric

/ required = true

</textbox>

<textbox vigorous_m>

/ caption = "Number of minutes per day:"

/ textboxsize = (10, 5)

/ fontstyle = ("Arial", 2.5%, false, false, false, false, 5, 1)

/ mask = alphanumeric

/ required = true

</textbox>

<textbox moderate_d>

/ caption = "Think about all the MODERATE activities that you did in the LAST 7 DAYS. Moderate activities refer to activities that take moderate physical effort and make you breathe somewhat harder than normal.

During the last 7 days, on how many days did you do moderate physical activities like carrying light loads, bicycling at a regular pace, or doubles tennis? Do not include walking.

Number of days per week: "

/ textboxsize = (10, 5)

/ fontstyle = ("Arial", 2.5%, false, false, false, false, 5, 1)

/ mask = alphanumeric

/ required = true

</textbox>

</textbox>

<textbox moderate_m>

/ caption = "Number of minutes per day:"

/ textboxsize = (10, 5)

/ fontstyle = ("Arial", 2.5%, false, false, false, false, 5, 1)

/ mask = alphanumeric

/ required = true

</textbox>

<textbox sedentary_d>

/ caption = "The last question is about the time you spent SITTING during the LAST 7 DAYS. Include time spent at work, at home, while doing course work and during leisure time.

This may include time spent sitting at a desk, visiting friends, reading, or sitting or lying down to watch television.

During the last 7 days, how much time did you spend sitting?

Number of days per week: "

/ textboxsize = (10, 5)

/ fontstyle = ("Arial", 2.5%, false, false, false, false, 5, 1)

/ mask = alphanumeric

/ required = true

</textbox>

<textbox sedentary_m>

/ caption = "Number of minutes per day:"

/ textboxsize = (10, 5)

/ fontstyle = ("Arial", 2.5%, false, false, false, false, 5, 1)

/ mask = alphanumeric

/ required = true

</textbox>

* Intention

<radiobuttons intention>

/ caption = "How much do you agree with following statement:

Over the next 7 days,

I intend to do at least 150 minutes of moderate-intensity physical activity;

or at least 75 minutes of vigorous intensity physical activity;

or an equivalent combination of moderate- and vigorous-intensity physical activity."

/ options = ("1~nStrongly ~nDisagree","2~n~n","3~n~n","4~n~n","5~n~n","6~n~n","7~nStrongly ~nAgree")

/ optionvalues = ("1", "2", "3", "4", "5", "6", "7")

/ fontstyle = ("Arial", 2.5%, false, false, false, false, 5, 1)

/ orientation = horizontalequal

/ required = true

</radiobuttons>

* Explicit affective attitude

<radiobuttons attitude1>

/ caption = "For me, to participate in regular physical activity is ..."

/ options = ("1~nUnpleasant","2~n~n","3~n~n","4~n~n","5~n~n","6~n~n","7~nPleasant")

/ optionvalues = ("1", "2", "3", "4", "5", "6", "7")

/ fontstyle = ("Arial", 2.5%, false, false, false, false, 5, 1)

/ orientation = horizontalequal

/ required = true

</radiobuttons>

<radiobuttons attitude2>

/ caption = "For me, to participate in regular physical activity is ..."

/ options = ("1~nUnenjoyable","2~n~n","3~n~n","4~n~n","5~n~n","6~n~n","7~nEnjoyable")

/ optionvalues = ("1", "2", "3", "4", "5", "6", "7")

/ fontstyle = ("Arial", 2.5%, false, false, false, false, 5, 1)

/ orientation = horizontalequal

/ required = true

</radiobuttons>

*************

Randomization

*************

<expt>

/subjects = (1 of 4)

/groupassignment = random

/ groups = (1 of 4)

/ blocks = [

1 = Consent;

2 = participant;

3 = demographics;

4 = approach_circle;

5 = reminder;

6 = images;

7 = approach_ap;

8 = reminder;

9 = images;

10 = avoid_ap;

11 = reminder;

12 = avoid_circle;

13 = survey

]

/ preinstructions = (intro)

/ postinstructions = (end)

/ onexptend = [values.completed = 1]

</expt>

<expt>

/subjects = (2 of 4)

/ groups = (2 of 4)

/groupassignment = random

/ blocks = [

1 = Consent;

2 = participant;

3 = demographics;

4 = avoid_circle;

5 = reminder;

6 = images;

7 = avoid_ap;

8 = reminder;

9 = images;

10 = approach_ap;

11 = reminder;

12 = approach_circle;

13 = survey

]

/ preinstructions = (intro)

/ postinstructions = (end)

/ onexptend = [values.completed = 1]

</expt>

<expt>

/subjects = (3 of 4)

/groupassignment = random

/ groups = (3 of 4)

/ blocks = [

1 = Consent;

2 = participant;

3 = demographics;

4 = avoid_circle;

5 = reminder;

6 = images;

7 = approach_ap;

8 = reminder;

9 = images;

10 = avoid_ap;

11 = reminder;

12 = approach_circle;

13 = survey

]

/ preinstructions = (intro)

/ postinstructions = (end)

/ onexptend = [values.completed = 1]

</expt>

<expt>

/subjects = (4 of 4)

/ groups = (4 of 4)

/groupassignment = random

/ blocks = [

1 = Consent;

2 = participant;

3 = demographics;

4 = approach_circle;

5 = reminder;

6 = images;

7 = avoid_ap;

8 = reminder;

9 = images;

10 = approach_ap;

11 = reminder;

12 = avoid_circle;

13 = survey

]

/ preinstructions = (intro)

/ postinstructions = (end)

/ onexptend = [values.completed = 1]

</expt>
