# Supplementary material for: Automatic approach-avoidance tendency toward physical activity, sedentary, and neutral stimuli as a function of age, explicit affective attitude, and intention to be active
Source: Peer Community J. Author manuscript; Available in PMC 2024 Dec 10. (PMC7617180; doi:10.24072/pcjournal.246)
Supplement: Data, code, and supplemental material [file EMS201322-supplement-Data__code__and_supplemental_material.zip › Boisgontier-Lab-Aging_Approach-Avoid_Physical-Activity-0a8d854/Materials/Consent_Form_EN.html]

 

**Consent Form**

Please read this consent document carefully before you decide to participate in this study. 

 

**Title of the study:** Automatic processes associated with physical activity and sedentary behavior from a lifespan perspective

**Purpose of the Study:** The purpose of the study is to improve our understanding of the mechanisms underlying the engagement in physical activity across the lifespan.

**Participation:** My participation in this 30-min online study will consist of responding to questionnaires and performing a reaction-time task on my computer using the Inquisit software. Questionnaires include questions related to demographic information, self-control resources, weight perception, body satisfaction, and apathy. In the reaction-time task, I will be asked to move a manikin toward or away from images presented on my computer screen by pressing keys on the keyboard.

 

**Risks:** My participation in this study will not cause me any risk or discomfort other than those of everyday life. However, if I feel any discomfort during the experiment, I may stop at any time by pressing the keys "ctrl + Q" to leave the experiment.

**Benefits:** My participation in this study will contribute to better understand the factors that facilitate or inhibit the engagement in physical activity, thereby helping individuals to adopt a more active and healthier lifestyle. My participation in this project will also help masters’ students in Physiotherapy at the University of Ottawa to develop their knowledge and skills in scientific research. 

 

**Confidentiality and Privacy:** My anonymity will be guaranteed by a unique confidential identification code.My responses to the questionnaires will remain strictly confidential. My name and contact information will not be collected, and my responses are therefore anonymous.

 

**Conservation of Data:** The data collected through the Inquisit website will be stored in world-class data center facilities of Inquisit in Oregon, USA and will not be moved to other locations. During data analyses, data will be stored on the University of Ottawa Microsoft OneDrive account of the principal investigator which is protected by a two-factor authentication.

**Voluntary Participation**: I am under no obligation to participate and if I choose to participate, I can withdraw from the study at any time and refuse to answer any questions, without suffering any negative consequences. If I choose to withdraw, I can contact the principal investigator and ask that the data related to my unique confidential identification code be removed. All data gathered until the time of withdrawal will then be removed from the dataset and not used in the study*.*

**Agreement:** I have read the above description of the study and I voluntarily agree to participate.

If I have any questions about the study, I may contact the principal investigator of the project via the contact information below. If I have any questions regarding the ethical conduct of this study, I may contact the Office of Research Ethics and Integrity via email (ethics@uottawa.ca) or telephone (613-562-5387). To get a copy of this consent and get additional information about my rights as a research participant in the study, I can email Dr. Matthieu Boisgontier (matthieu.boisgontier@uottawa.ca) at the School of Rehabilitation Sciences, Faculty of Health Sciences, University of Ottawa. Participants are encouraged to save a copy of the consent form.

 

**Acceptance:** By selecting the consent statement below, I agree or disagree to participate in this research study.

 

 
