# Supplementary figures and images for: Automatic approach-avoidance tendency toward physical activity, sedentary, and neutral stimuli as a function of age, explicit affective attitude, and intention to be active
Source: Peer Community J. Author manuscript; Available in PMC 2024 Dec 10. (PMC7617180; doi:10.24072/pcjournal.246)

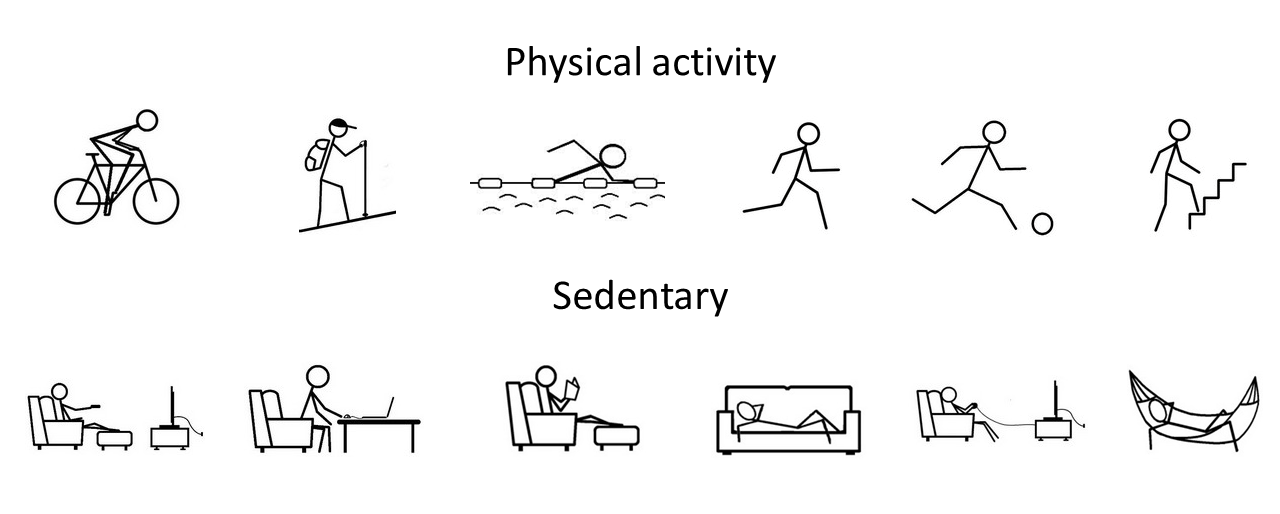

Supplement: Data, code, and supplemental material [file EMS201322-supplement-Data__code__and_supplemental_material.zip › Boisgontier-Lab-Aging_Approach-Avoid_Physical-Activity-0a8d854/Materials/instructionimages.png]

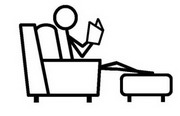

Supplement: Data, code, and supplemental material [file EMS201322-supplement-Data__code__and_supplemental_material.zip › Boisgontier-Lab-Aging_Approach-Avoid_Physical-Activity-0a8d854/Materials/stimuli/SED-LECT.jpg]

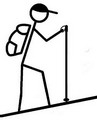

Supplement: Data, code, and supplemental material [file EMS201322-supplement-Data__code__and_supplemental_material.zip › Boisgontier-Lab-Aging_Approach-Avoid_Physical-Activity-0a8d854/Materials/stimuli/AP-RANDO.jpg]

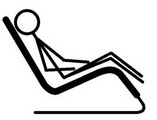

Supplement: Data, code, and supplemental material [file EMS201322-supplement-Data__code__and_supplemental_material.zip › Boisgontier-Lab-Aging_Approach-Avoid_Physical-Activity-0a8d854/Materials/stimuli/SED-CHAISE.jpg]

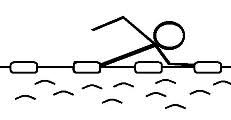

Supplement: Data, code, and supplemental material [file EMS201322-supplement-Data__code__and_supplemental_material.zip › Boisgontier-Lab-Aging_Approach-Avoid_Physical-Activity-0a8d854/Materials/stimuli/AP-NAT.jpg]

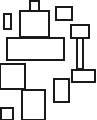

Supplement: Data, code, and supplemental material [file EMS201322-supplement-Data__code__and_supplemental_material.zip › Boisgontier-Lab-Aging_Approach-Avoid_Physical-Activity-0a8d854/Materials/stimuli/AP-RANDOc.jpg]

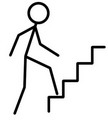

Supplement: Data, code, and supplemental material [file EMS201322-supplement-Data__code__and_supplemental_material.zip › Boisgontier-Lab-Aging_Approach-Avoid_Physical-Activity-0a8d854/Materials/stimuli/AP-ESCAL.jpg]

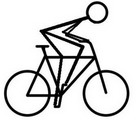

Supplement: Data, code, and supplemental material [file EMS201322-supplement-Data__code__and_supplemental_material.zip › Boisgontier-Lab-Aging_Approach-Avoid_Physical-Activity-0a8d854/Materials/stimuli/AP-VEL.jpg]

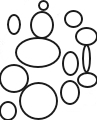

Supplement: Data, code, and supplemental material [file EMS201322-supplement-Data__code__and_supplemental_material.zip › Boisgontier-Lab-Aging_Approach-Avoid_Physical-Activity-0a8d854/Materials/stimuli/AP-RANDOr.jpg]

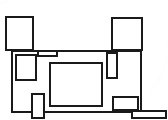

Supplement: Data, code, and supplemental material [file EMS201322-supplement-Data__code__and_supplemental_material.zip › Boisgontier-Lab-Aging_Approach-Avoid_Physical-Activity-0a8d854/Materials/stimuli/SED-HAMACc.jpg]

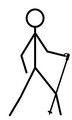

Supplement: Data, code, and supplemental material [file EMS201322-supplement-Data__code__and_supplemental_material.zip › Boisgontier-Lab-Aging_Approach-Avoid_Physical-Activity-0a8d854/Materials/stimuli/AP-MARCH.jpg]

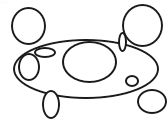

Supplement: Data, code, and supplemental material [file EMS201322-supplement-Data__code__and_supplemental_material.zip › Boisgontier-Lab-Aging_Approach-Avoid_Physical-Activity-0a8d854/Materials/stimuli/SED-HAMACr.jpg]

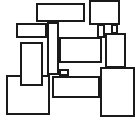

Supplement: Data, code, and supplemental material [file EMS201322-supplement-Data__code__and_supplemental_material.zip › Boisgontier-Lab-Aging_Approach-Avoid_Physical-Activity-0a8d854/Materials/stimuli/AP-VELc.jpg]

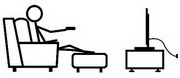

Supplement: Data, code, and supplemental material [file EMS201322-supplement-Data__code__and_supplemental_material.zip › Boisgontier-Lab-Aging_Approach-Avoid_Physical-Activity-0a8d854/Materials/stimuli/SED-TV.jpg]

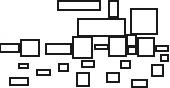

Supplement: Data, code, and supplemental material [file EMS201322-supplement-Data__code__and_supplemental_material.zip › Boisgontier-Lab-Aging_Approach-Avoid_Physical-Activity-0a8d854/Materials/stimuli/AP-NATc.jpg]

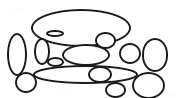

Supplement: Data, code, and supplemental material [file EMS201322-supplement-Data__code__and_supplemental_material.zip › Boisgontier-Lab-Aging_Approach-Avoid_Physical-Activity-0a8d854/Materials/stimuli/SED-CANAPr.jpg]

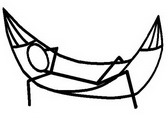

Supplement: Data, code, and supplemental material [file EMS201322-supplement-Data__code__and_supplemental_material.zip › Boisgontier-Lab-Aging_Approach-Avoid_Physical-Activity-0a8d854/Materials/stimuli/SED-HAMAC.jpg]

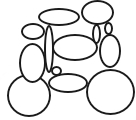

Supplement: Data, code, and supplemental material [file EMS201322-supplement-Data__code__and_supplemental_material.zip › Boisgontier-Lab-Aging_Approach-Avoid_Physical-Activity-0a8d854/Materials/stimuli/AP-VELr.jpg]

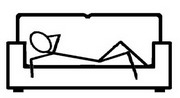

Supplement: Data, code, and supplemental material [file EMS201322-supplement-Data__code__and_supplemental_material.zip › Boisgontier-Lab-Aging_Approach-Avoid_Physical-Activity-0a8d854/Materials/stimuli/SED-CANAP.jpg]

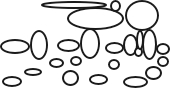

Supplement: Data, code, and supplemental material [file EMS201322-supplement-Data__code__and_supplemental_material.zip › Boisgontier-Lab-Aging_Approach-Avoid_Physical-Activity-0a8d854/Materials/stimuli/AP-NATr.jpg]

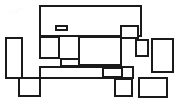

Supplement: Data, code, and supplemental material [file EMS201322-supplement-Data__code__and_supplemental_material.zip › Boisgontier-Lab-Aging_Approach-Avoid_Physical-Activity-0a8d854/Materials/stimuli/SED-CANAPc.jpg]

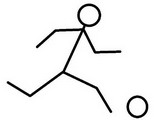

Supplement: Data, code, and supplemental material [file EMS201322-supplement-Data__code__and_supplemental_material.zip › Boisgontier-Lab-Aging_Approach-Avoid_Physical-Activity-0a8d854/Materials/stimuli/AP-FOOT.jpg]

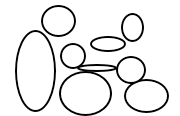

Supplement: Data, code, and supplemental material [file EMS201322-supplement-Data__code__and_supplemental_material.zip › Boisgontier-Lab-Aging_Approach-Avoid_Physical-Activity-0a8d854/Materials/stimuli/SED-LECTr.jpg]

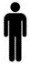

Supplement: Data, code, and supplemental material [file EMS201322-supplement-Data__code__and_supplemental_material.zip › Boisgontier-Lab-Aging_Approach-Avoid_Physical-Activity-0a8d854/Materials/stimuli/manikin_2.jpg]

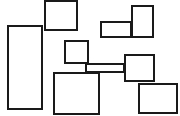

Supplement: Data, code, and supplemental material [file EMS201322-supplement-Data__code__and_supplemental_material.zip › Boisgontier-Lab-Aging_Approach-Avoid_Physical-Activity-0a8d854/Materials/stimuli/SED-LECTc.jpg]

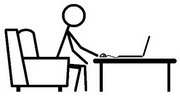

Supplement: Data, code, and supplemental material [file EMS201322-supplement-Data__code__and_supplemental_material.zip › Boisgontier-Lab-Aging_Approach-Avoid_Physical-Activity-0a8d854/Materials/stimuli/SED-ORDI.jpg]

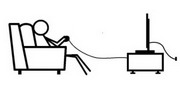

Supplement: Data, code, and supplemental material [file EMS201322-supplement-Data__code__and_supplemental_material.zip › Boisgontier-Lab-Aging_Approach-Avoid_Physical-Activity-0a8d854/Materials/stimuli/SED-JVID.jpg]

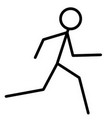

Supplement: Data, code, and supplemental material [file EMS201322-supplement-Data__code__and_supplemental_material.zip › Boisgontier-Lab-Aging_Approach-Avoid_Physical-Activity-0a8d854/Materials/stimuli/AP-COUR.jpg]
